# Solvolysis Artifacts: Leucettazoles as Cryptic Macrocyclic Alkaloid Dimers from a Southern Australian Marine Sponge, *Leucetta* sp.

**DOI:** 10.3390/md17020106

**Published:** 2019-02-09

**Authors:** Pritesh Prasad, Angela A. Salim, Shamsunnahar Khushi, Zeinab G. Khalil, Michelle Quezada, Robert J. Capon

**Affiliations:** Institute for Molecular Bioscience, The University of Queensland, St Lucia, QLD 4072, Australia; p.prasad@imb.uq.edu.au (P.P.); a.salim@uq.edu.au (A.A.S.); s.khushi@imb.uq.edu.au (S.K.); z.khalil@imb.uq.edu.au (Z.G.K.); michelle.quezada@newcastle.edu.au (M.Q.)

**Keywords:** ethanolysis, solvolysis, artifact, leucettazoles, leucettazines, macrocyclic alkaloids, *Leucetta*, Australian sponge, GNPS

## Abstract

Chemical analysis of a southern Australian sponge, *Leucetta* sp., led to the discovery of a pair of solvolysis adducts, leucettazoles A1 (**1a**) and B1 (**2a**), as artifacts of an unprecedented family of 15-membered macrocyclic alkaloid dimers featuring a pair of imino bridged 2-aminoimidazoles, together with a putative monomeric precursor, leucettazine A (**3**). The dimeric alkaloids **1a** and **2a**, and monomer **3**, were identified by detailed spectroscopic analysis, supported by chemical transformations, analytical mass spectrometry, and biosynthetic considerations. Global natural product social networking (GNPS) molecular analysis of crude sponge extracts and solvent partitions, supported by single ion extraction (SIE) and diagnostic MS/MS fragmentations, revealed the associated natural products, leucettazoles A (**1**) and B (**2**). This study highlights that the study of natural product artifacts can be useful, and can on occasion serve as a pathway to discover cryptic new classes of natural products.

## 1. Introduction

Marine sponges are a remarkable source of structurally diverse natural products. For example, over several decades, our investigation of southern Australian marine sponges revealed a wealth of natural product classes, many unique to marine sponges. These include the terpenyls luffarins [1] and trunculins [2,3,4], the alkaloids trachycladindoles [5] and dragmacidins [6], the polyketides franklinolides [7] and amphilactams [8], the lipids heterofibrins [9] and thiocyanatins [10,11], and the carbohydrate 5-thiomannose [12].

During our studies into sponges, and other Australian marine biodiversity, we regularly encounter natural products that are prone to structural transformation during extraction, isolation, and/or handling. Such compounds, typically denoted as artifacts, enjoy a questionable presence in the field of natural products. For example, where some natural product chemists tend to ignore artifacts, opting instead to describe every isolated compound as a natural product, others are willing to acknowledge the presence of artifacts, but nevertheless relegate them to inconsequential footnotes. In contrast to both these somewhat dismissive stances, we share a deeper appreciation of artifacts, in common with those of our colleagues who recognize, document, report on, and exploit knowledge of their chemical and biological properties. Appreciating the potential value of artifacts served us, and others, very well. For example, in our hands, they informed our understanding of many rare and unique structure classes, including (i) configurationally and solvolytically unstable anticancer polyketide phosphodiester franklinolides, isolated from a *Halichondria*/*Geodia* sp. sponge complex [7]; (ii) the solvolytically reactive P-glycoprotein inhibitory diketomorpholine shornephine A, isolated from a marine sediment-derived *Aspergillus* sp. CMB-M081F [13]; (iii) a family of hydrazine Schiff base prolinimines, recovered from cultivations of a marine fish gastrointestinal tract-derived *Trichoderma* sp. CMB-F563 [14]; (iv) acid-mediated intramolecular cycloaddition products derived from macrocyclic polyketide cytochalasins, isolated from a marine sediment-derived *Phomopsis* sp. CMB-M0042F [15]; (v) interconverting spiroketal–polyketide antifungal reveromycins from both marine and terrestrial *Streptomyces* spp. MST-MA568 and MST-RA7781 [16]; and (vi) oxygen- and light-mediated cycloaddition products from polyene macrolactams, heronamides, isolated from a marine beach sand-derived *Streptomyces* sp. CMB-M0406 [17,18]. Building on prior experience, this current report demonstrates how the knowledge of solvolysis artifacts isolated from a southern Australian marine sponge, *Leucetta* sp. (CMB-01047), informed the discovery of an unprecedented family of macrocyclic alkaloid dimers.

## 2. Results and Discussion

**Figure 1:**
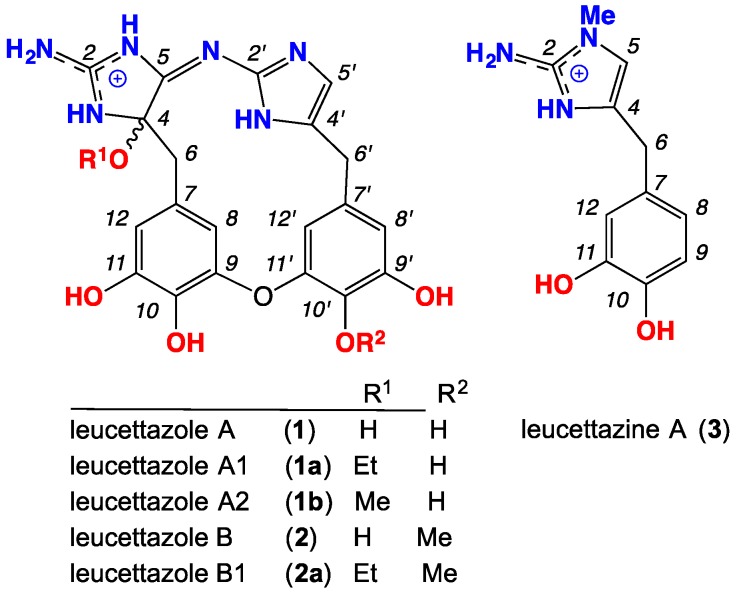
Chemistry of *Leucetta* sp. (CMB-01047) (as trifluoroacetate (TFA) salts).

A portion of the aqueous ethanol (EtOH) extract of a sponge sample *Leucetta* sp. (CMB-01047) was decanted, concentrated in vacuo, and subjected to a sequence of solvent partitions and triturations, followed by reversed-phase HPLC, to yield leucettazoles A1 (**1a**) and B1 (**2a**), and leucettazine A (**3**) (Figure 1). The structure elucidation of **1a**, **2a**, and **3** was achieved by detailed spectroscopic analysis, supported by biosynthetic considerations and chemical transformation. The latter included *trans*-solvolysis of **1a** in methanol to leucettazole A2 (**1b**). With **1a** and **2a** designated as ethanolysis adducts (i.e., artifacts), the putative natural product leucettazoles A (**1**) and B (**2**) were detected and identified in the crude aqueous EtOH extract and *n*-butanol (*n*-BuOH) partitions by a combination of (i) ultra-high-performance liquid chromatography/quadrupole time-of-flight (UPLC-QTOF) analysis with single ion extraction (SIE), (ii) global natural product social (GNPS) molecular networking, and (iii) MS/MS analysis. Identification of leucettazole A (**1**) was further confirmed by spectroscopic analysis of a small, mixed 3:1 sample of **1** and **3**.

### 2.1. Leucettazole A1 *(**1a**)*

**Figure 2:**
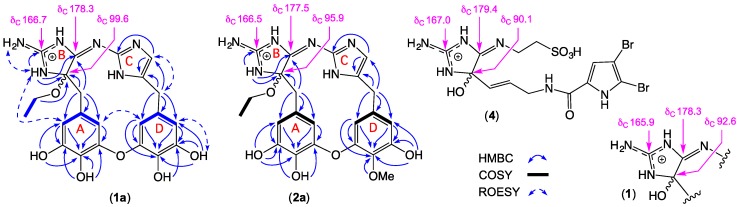
Selected NMR (dimethyl sulfoxide, DMSO-*d*_6_) data for leucettazoles A (**1**), A1 (**1a**), and B1 (**2a**), and tauroascidin E (**4**) (as TFA salts).

High-resolution electrospray ionization mass spectrometry (HRESI(+)MS) analysis of **1a** revealed a molecular ion attributed to a salt with a molecular formula (C_22_H_23_N_6_O_6_^+^, ∆mmu −0.3) incorporating 15 double-bond equivalents (DBE). The ^1^H NMR (dimethyl sulfoxide, DMSO-*d*_6_) data for **1a** (Table 1; Figure S1, Supplementary Materials) revealed resonances attributed to a diastereotopic OEt moiety (OCH_2_CH_3_ δ_H_ 3.31, dq and 3.45, dq). Although this diastereotopic character was suggestive of proximity to a chiral center, the absence of an optical rotation required that **1a** be racemic. This, together with long-term storage in EtOH, alerted us to the possibility that **1a** was an ethanolysis adduct (i.e., an artifact). The two-dimensional (2D) NMR data for **1a** (Table 1 and Figure 2; Figures S3–S6, Supplementary Materials) also established the presence of two 3,4,5-trioxybenzyl subunits (rings A and B), with diagnostic HMBC correlations positioning phenol moieties: 10-OH, (δ_H_ 8.68), 11-OH (δ_H_ 8.87), 9′-OH (δ_H_ 9.29), and 10′-OH (δ_H_ 8.86). A ROESY correlation between H-8 and H-12′ supported a C-9 to C-10′ ether bridge. These assignments were supported by COSY correlations between H-8 and H-12, and H-8′ and H-12′, together with *J*_8,12_ and *J*_8′,12′_ meta (2.0 Hz) coupling.

To account for the remaining C_8_H_10_N_6_ and seven DBE, we speculated that **1a** incorporated two 2-aminoimidazoles (rings C and B). Analysis of ^1^H–^15^N HSQC NMR (DMSO-*d*_6_) data for **1a** (Figure S7) confirmed the presence of 3-NH (δ_H_ 9.16; δ_N_ 103.6) and 2-NH_2_ (δ_H_ 8.02 and 8.23; δ_N_ 87.5), while a ROESY correlation between these two moieties, along with HMBC correlations from (i) 3-NH to C-2 (δ_C_ 166.7), C-4 (δ_C_ 96.6), and C-5 (δ_C_ 178.3), (ii) H_2_-6 to C-4 and C-5, and (iii) the OEt to C-4, permitted assembly of ring B, including linkage to ring A (Figure 2). To the best of our knowledge, the only reported example of a compound incorporating a comparable ring B heterocycle is tauroascidin E (**4**) (Figure 2), first described in 2015 from an Okinawan marine sponge, *Agelas* sp. [19]. Significantly, **1a** and **4** share comparable ^13^C NMR chemical shifts for the guanidine sp^2^ C-2, oxo-amino sp^3^ C-4, and imino sp^2^ C-5 resonances (Figure 2). The absence of any 2D NMR correlations to the exchangeable proton in ring C (δ_H_ 11.82) likely reflects facile equilibrium between 1′-NH and 3′- NH tautomers (arbitrarily represented as the 3′-NH tautomer).

Tautomerization notwithstanding, HMBC correlations from (i) H_2_-6′ to C-4′ and C-5′, and (ii) H-5′ to C-2′ and C-4′ (Figure 2) permitted assembly of the ring C heterocycle in **1a**, including linkage to ring D. Finally, to account for the molecular formula, rings B and C were linked via an imino bridge, thereby assembling the macrocyclic structure for leucettazole A1 (**1a**), as shown. Although **1a** was isolated as the trifluoroacetate (TFA) salt, the additional NH resonance was undetected, most likely due to tautomeric equilibrium (arbitrarily represented as the 3-NH tautomer). Of note, ROESY correlations between H-5′ and H-12′, between H-12′ and H-8, and between 3-NH and H-12 suggest that **1a** adopts a basket-like (rather than planar) configuration.

**Figure 3:**
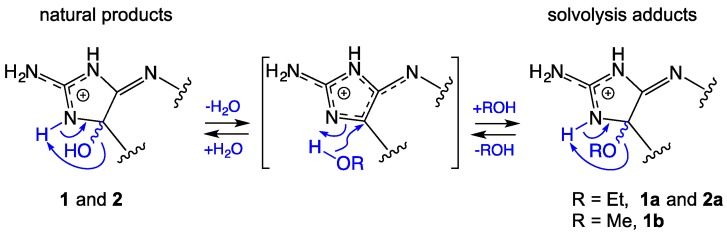
A plausible pathway for the reversible solvolysis of natural products **1** and **2**.

As noted above, the presence of a racemic tertiary ethoxy moiety in **1a** was taken as evidence that prolonged storage in EtOH induced ethanolysis of a putative natural product, leucettazole A (**1**). To test this hypothesis, separate samples of crude EtOH extract, and the pure ethoxy adduct **1a** were heated in sealed tubes at 40 °C and 60 °C, in either methanol (MeOH) or 50% MeCN/H_2_O. Consistent with our hypothesis, UHPLC-QTOF with single ion extraction (SIE) analysis of either the crude EtOH extract or pure **1a** treated with MeOH yielded a new peak attributed to a methanolysis adduct, leucettazole A2 (**1b**) (C_21_H_21_N_6_O_6_^+^, ∆mmu +0.1) (Figures S16–S19, Supplementary Materials). As expected, the MeOH adduct was not observed when either the crude EtOH extract or pure **1a** was treated with MeCN/H_2_O (Figures S20 and S21, Supplementary Materials). Interestingly, when treated with 0.02% TFA/H_2_O at 60 °C, **1a** regenerated the putative natural product **1** (Figures S22 and S23, Supplementary Materials). These observations are explained by the proposed solvolysis equilibrium pathway outlined in Figure 3.

### 2.2. Leucettazole B1 *(**2a**)*

HRESI(+)MS analysis of **2a** revealed a molecular ion attributed to a salt with a molecular formula (C_23_H_25_N_6_O_6_^+^, ∆mmu −0.2) consistent with a homolog (+CH_2_) of **1a**. Comparison of the one-dimensional (1D) and 2D NMR (DMSO-*d6*) data for **2a** (Table 2 and Figure 2; Figures S8 and S9, Supplementary Materials) with **1a** revealed the principle difference as a replacement of resonances for a phenol with those for a methoxy (δ_H_ 3.71; δ_C_ 59.9), with the C-10′ regiochemistry confirmed by an HMBC correlation from the OCH_3_ to C-10′. As with **1a**, the structure assigned to leucettazole B1 (**2a**) was suggestive of an ethanolysis adduct (Figure 3).

### 2.3. Leucettazine A *(**3**)*

**Figure 4:**
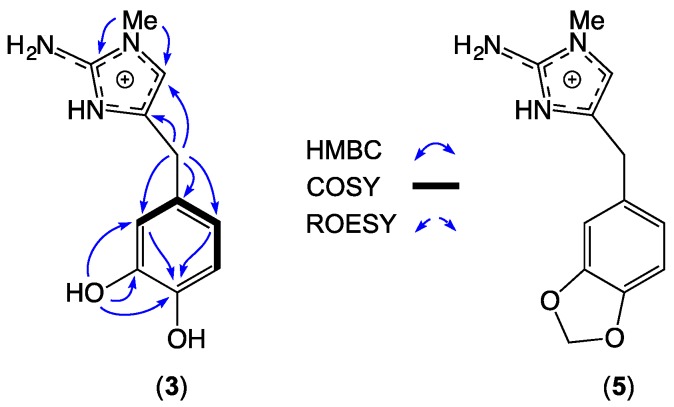
Selected NMR (DMSO-*d*_6_) data for leucettazine A (**3**) (as a TFA salt), and preclathridine A (**5**).

HRESI(+)MS analysis of **3** revealed a molecular ion attributed to a salt with a molecular formula (C_11_H_14_N_3_O_2_^+^, ∆mmu 0.0) requiring seven DBE, and consistent with a carbon homolog of the known *Leucetta* metabolite preclathridine A (**5**) [20]. Analysis of the 1D and 2D NMR (DMSO-*d*_6_) data for **3** (Table 3; Figures S10 and S11, Supplementary Materials), revealed D_2_O exchangeable resonances attributed to a 3-NH (*δ*_H_ 12.06), 11-OH (*δ*_H_ 8.82), 10-OH (*δ*_H_ 7.43), and 2-NH_2_, (*δ*_H_ 6.54), together with resonances for an imidazole H-5/C-5 (*δ*_H_ 6.61; *δ*_C_ 113.7) and 1-NMe (*δ*_H_ 3.37; *δ*_C_ 31.8), and a 3,4-dihydroxylbenzyl moiety (H-12, *δ*_H_ 6.59, d, 1.5 Hz; H-8, *δ*_H_ 6.47, dd, 8.0, 1.5 Hz; H-9, *δ*_H_ 6.66, d, 8.0 Hz; H_2_-6, *δ*_H_ 3.58, s). Diagnostic correlations in the 2D NMR data (Figure 4) permitted assembly of the structure for leucettazine A (**3**) as shown. Significantly, the 1-NMe regiochemistry was evident from HMBC correlations from the *N*-Me (*δ*_H_ 3.37) to both C-2 (*δ*_C_ 146.2) and C-5 (*δ*_C_ 113.7).

### 2.4. Visualization of Leucettazoles A *(**1**)* and B *(**2**)*

**Figure 5:**
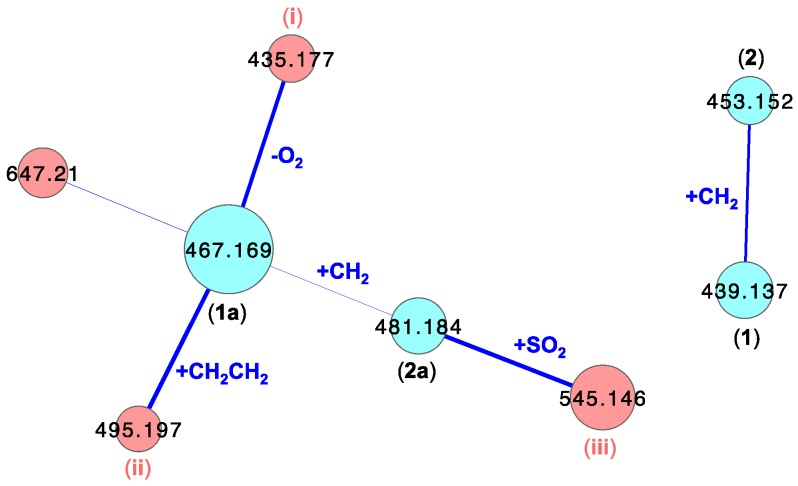
Leucettazole global natural product social (GNPS) clusters from *n*-butanol (*n*-BuOH) solubles of *Leucetta* sp. (CMB-01047).

**Figure 6:**
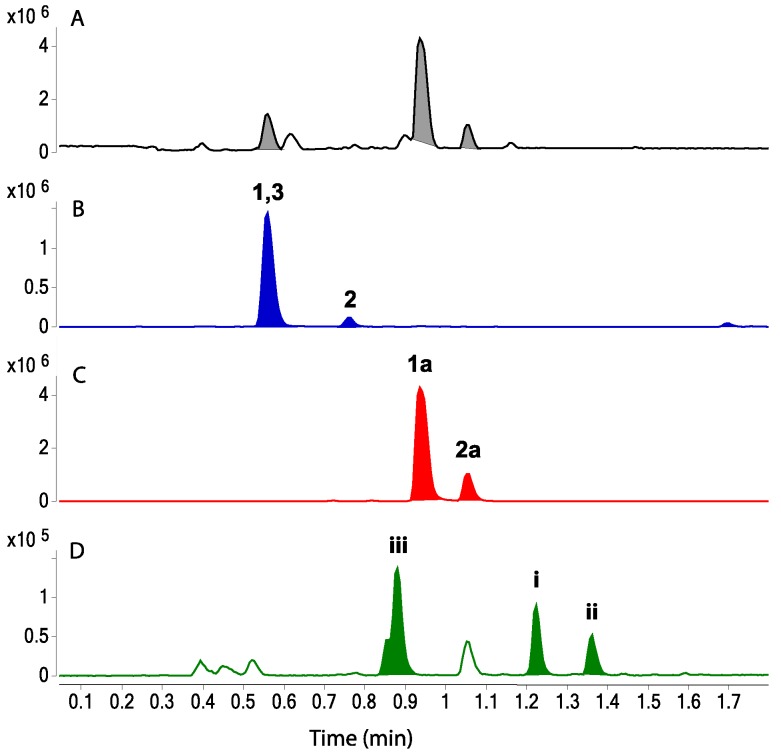
Ultra-high-performance liquid chromatography/quadrupole time-of-flight (UPLC-QTOF) analysis of *n*-BuOH solubles; (**A**) total ion chromatogram, with single ion extraction (SIE) chromatograms for (**B**) *m/z* 439 (**1**), 453 (**2**), and 220 (**3**); (**C**) *m/z* 467 (**1a**) and 481 (**2a**); and (**D**) *m/z* 435 (**i**), 495 (**ii**), and 545 (**iii**).

To visualize leucettazole’s chemical diversity in *Leucetta* sp. (CMB-01047), we acquired UHPLC-QTOF-MS/MS data on the unfractionated *n*-BuOH solubles, and subjected it to a global natural product social (GNPS) molecular networking analysis [21]. This analysis revealed a cluster incorporating both ethanolysis adducts **1a** and **2a**, along with minor components tentatively attributed, on the basis of cosine scores and accurate mass measurements, as a (**i**) dideoxy (−32 Da, C_22_H_23_N_6_O_4_^+^, ∆mmu +1.0) and (**ii**) *n*-butanol solvolysis (+28 Da, exchange of EtOH for *n*-BuOH, C_24_H_27_N_6_O_6_^+^, ∆mmu −3.5), analogs of **1a**, and (**iii**) a sulfonated (+64 Da, C_23_H_25_N_6_O_8_S^+^, ∆mmu −1.5) analog of **2a** (Figure 5). Of even greater significance, GNPS analysis revealed a cluster incorporating the putative natural products, leucettazoles A (**1**) (C_20_H_19_N_6_O_6_^+^, ∆mmu +0.9) and B (**2**) (C_21_H_21_N_6_O_6_^+^, ∆mmu +0.3). UHPLC-QTOF-SIE analysis of the *n*-BuOH solubles (Figure 6), and analysis of MS/MS fragmentations (Figures S24–S30, Supplementary Materials), further validated the presence of the natural products **1** and **2**, and solvolysis adducts **1a** and **2a** (and the species **i**–**iii**).

### 2.5. Leucettazole A *(**1**)*

Although leucettazoles A (**1**) and B (**2**) were detected in the crude extract (see above), neither could be isolated in a pure form. Notwithstanding, spectroscopic analysis of a small quantity of a 3:1 mixed sample of **1** and **3** did provide valuable supporting evidence. More specifically, excellent concordance between the 1D NMR (DMSO-d_6_) data for **1** with **1a** (Table 4; Figures S12–S15, Supplementary Materials), and for key ^13^C NMR resonances with **1a**, **2a**, and **4** (Figure 2) supported assignment of the structure for leucettazole A (**1**) as shown.

### 2.6. Leucettazole Biology

Monomeric 2-aminoimidazoles recovered from *Leucetta* spp. are attributed a range of biological activities including mammalian cytotoxicity [22] and leukotriene B4 (LTB4) receptor antagonism with possible anti-inflammatory potential [23], and inspired synthetic analogs (i.e., leucettines) that exhibit selective kinase inhibitory activity with potential application against Alzheimer’s disease and Down syndrome [24]. In our hands, the leucettazoles A1 (**1a**) and B1 (**2a**) did not exhibit growth inhibitory activity (i.e., half maximal inhibitory concentration (IC_50_) > 30 μM) against the Gram-negative bacteria *Escherichia coli* American Type Culture Collection (ATCC) 25922 and *Pseudomonas aeruginosa* ATCC 10145, the Gram-positive bacteria *Staphylococcus aureus* ATCC 25923 and *Bacillus subtilis* ATCC 6633, the fungus *Candida albicans* ATCC 10231, on human colorectal (SW620) or embryonic kidney (HEK293) carcinoma cells.

### 2.7. Leucettazole Biosynthesis

**Figure 7:**
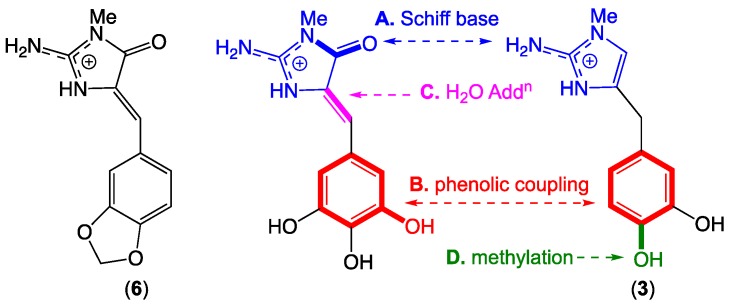
Plausible leucettazole biosynthesis (and biomimetic synthesis), and structure for leucettamine B.

A plausible biosynthesis of the leucettazoles could proceed as outlined in Figure 7. In this hypothesis, leucettazine A (**3**) dimerizes with an oxidized analog via (**A**) a Schiff base and (**B**) phenolic coupling, accompanied by (**C**) C-4 H_2_O addition, to yield the leucettazole macrocycle, with (**D**) regiospecific 10′-OH methylation occurring pre- or post-dimerization. Supportive of this hypothesis, a Palauan marine sponge *Leucetta microraphis* was reported to yield a comparable oxidized analog in the form of leucettamine B (**6**) (Figure 7) [23].

## 3. Materials and Methods

### 3.1. General Experimental Procedures

Specific optical rotation ([α]_D_) measurements were acquired on a JASCO P-1010 polarimeter in a 100 × 2 mm cell at room temperature (22 °C). Ultraviolet–visible (UV–Vis) spectra were obtained on a Cary 50 UV–visible spectrophotometer. NMR spectra were obtained on a Bruker Avance DRX600 spectrometer equipped with either a 5-mm PASEL ^1^H/D-^13^C Z-Gradient probe or 5-mm CPTCI ^1^H/^19^F-^13^C/^15^N/DZ-Gradient cryoprobe, controlled by TopSpin 2.1 software. Chemical shifts (ppm) were referenced internally against residual solvent signals (DMSO-*d*_6_: *δ*_H_ 2.50, *δ*_C_ 39.5). High-resolution electrospray ionization mass spectra (HRESIMS) were obtained on a Bruker micrOTOF mass spectrometer by direct infusion in MeCN at 3 μL/min using sodium formate clusters as an internal calibrant. HPLC–MS data were acquired using an Agilent 1100 series separation module equipped with a diode-array multiple wavelength detector coupled to an Agilent 1100 series LC/mass selective detector (MSD), operating in positive and negative modes using electrospray ionization (ESI) mode. Ultra-high-performance liquid chromatography (UHPLC) was performed on an Agilent 1290 infinity UHPLC system composed of a 1290 infinity quaternary pump, thermostat, autosampler, and photodiode-array detector. Analytical, semi-preparative, and preparative HPLC analyses were performed on Agilent 1100 series LC modules, with corresponding detectors and fraction collectors. All solvents used for HPLC separation and purification were chromatographic grade.

### 3.2. Collection and Taxonomy

A specimen of marine sponge, *Leucetta* sp. (CMB-01047), was collected in 1990 by hand (SCUBA) from submerged shallow water rock platforms off Flinders, Victoria, Australia. The sponge material was cooled to 0 °C shortly after collection, and transported to the laboratory, where it was documented, diced, and steeped in EtOH at −20 °C for long-term storage. The taxonomic description of the specimen is as follows: *Leucetta* sp. (class, Calcarea; order, Clathrinida; family, Leucettidae); growth form—irregular massive; texture—harsh friable; oscules—small scattered; surface—translucent porous; spicules—triactines regular, equiangular, size range (rays 10–120 μm); ectosome—indistinct from choanosome; choanosome—leuconoid, densely spiculose, lacunose, little collagen, exclusively free spicules, solid body; color in EtOH—brown. A voucher sample was deposited with Museum Victoria (Registry No. MVF222666).

### 3.3. Extraction and Isolation

A portion (300 mL) of the crude aqueous EtOH extract of *Leucetta* sp. CMB-01047 was decanted, concentrated in vacuo, and partitioned between H_2_O and *n*-BuOH. The *n*-BuOH-soluble material (731.2 mg) was concentrated in vacuo, subjected to sequential solvent trituration, and concentrated in vacuo to yield materials soluble in *n*-hexane (42.0 mg), CH_2_Cl_2_ (21.9 mg), and MeOH (270.4 mg). The MeOH solubles were subjected to preparative HPLC (Agilent Zorbax SB-C_8_ 21.2 × 150 mm, 5-μm column, 20.0 mL/min, 14.0-min linear-gradient elution from 10% CH_3_CN/H_2_O to 32% CH_3_CN/H_2_O with a constant 0.01% TFA modifier, followed by isocratic elution at 32% CH_3_CN/H_2_O (+0.01% TFA) for 3.0 min) to afford leucettazole A1 (**1a**) (2.0 mg, 0.042%), leucettazole B1 (**2a**) (1.6 mg, 0.033%), and a mixture containing leucettazole A (**1**) and leucettazine A (**3**) (3.5 mg). Fractionation of the latter mixture by semi-preparative HPLC (Agilent Zorbax SB-C_8_ 9.4 × 250 mm, 5-μm column, 3.5 mL/min, 14.0-min linear-gradient elution from 10% CH_3_CN/H_2_O to 14% CH_3_CN/H_2_O with a constant 0.01% TFA modifier) yielded pure leucettazine A (**3**) (0.6 mg, 0.013%). Note that purification using reverse-phase HPLC utilizing TFA as a modifier yielded compounds as TFA salts. The percentage yields were calculated as the estimated weight/weight percentage of each compound in the crude EtOH extract.

*Leucettazole A* (**1**): light-yellow powder, as a mixture with **3**; UV (MeCN) λ_max_ 318 nm, 284 nm; 1D and 2D NMR (DMSO-*d*_6_), see Table 4 and Figures S12–S15 (Supplementary Materials); HRESI(+)MS *m/z* 439.1361 (calculated for C_20_H_19_N_6_O_6_^+^, 439.1361).

*Leucettazole A1* (**1a**): light-yellow powder; [α]_D_^22^ 0.0 (*c* 0.8, MeOH); UV (MeOH) λ_max_ 318 nm (ε 56293), 284 nm (ε 4335); 1D and 2D NMR (DMSO-*d*_6_), see Table 1 and Figures S1 and S2 (Supplementary Materials); HRESI(+)MS *m/z* 467.1677 (calculated for C_22_H_23_N_6_O_6_^+^, 467.1674).

*Leucettazole B1* (**2a**): light-yellow powder; [α]_D_^22^ 0.0 (*c* 0.8, MeOH); UV (MeOH) λ_max_ 318 nm (ε 56295), 284 nm (ε 4725); 1D and 2D NMR (DMSO-*d*_6_), see Table 2 and Figures S8 and S9 (Supplementary Materials); HRESI(+)MS *m/z* 481.1832 (calculated for C_23_H_25_N_6_O_6_^+^, 481.1830).

*Leucettazine A* (**3**): light-yellow powder; 1D and 2D NMR (DMSO-*d*_6_), see Table 3 and Figures S10 and S11 (Supplementary Materials); HRESI(+)MS *m/z* 220.1084 (calculated for C_11_H_14_N_3_O_2_^+^, 220.1081).

### 3.4. Crude EtOH Extract Solvolysis Studies

Individual aliquots (1.0 mL) of EtOH crude extract of *Leucetta* sp. (CMB-01047) were concentrated in vacuo, re-dissolved in MeOH, and heated overnight in sealed vials at 40 °C and 60 °C. MS/MS data were acquired by subjecting an aliquot of CMB-01047 crude extract (1 μL) to UHPLC-QTOF analysis. UHPLC conditions involved a 0.5-mL/min gradient elution from 10% CH_3_CN/H_2_O to 100% CH_3_CN over a period of 4.5 min, with constant 0.1% formic acid, through an Agilent SB-C_8_ 1.8-μm, 2.1 × 50 mm column with column compartment temperature set at 40 °C. The source parameters were as follows: electrospray positive ionization; mass range of *m/z* 50–1700; MS scan rate, 3× per second; MS/MS scan rate, 3× per second; fixed collision energy, 40 eV; source gas temperature, 325 °C; gas flow, 10 L/min; and nebulizer, 20 psig. The scan source parameters were as follows: VCap 4000; fragmentor 180; skimmer 45; and octopole RF Peak 750. SIE at *m/z* 453.1500 (peak width ± 0.05 amu) revealed a species eluting at a retention time of 0.82 min (Figure S16, Supplementary Materials). Further analysis revealed a molecular ion attributed to a salt (*m/z* 453.1516) with a molecular formula (C_21_H_21_N_6_O_6_^+^, calculated 453.1517), consistent with the proposed structure for the methanolysis adduct leucettazole A2 (**1b**). MS/MS analysis of **1b** revealed diagnostic fragmentation consisting of the loss of OMe (*m/z* 421.1235) (Figure S17, Supplementary Materials).

### 3.5. Leucettazole A1 *(**1a**)* Solvolysis Studies

Individual aliquots (20 μg) of leucettazole A1 (**1a**) recovered from *Leucetta* sp. (CMB-01047) were dissolved in either MeOH, MeCN, or 0.02% TFA/H_2_O and heated (12 h) in sealed vials at 40 °C and 60 °C. An aliquot was subjected to UPLC-QTOF analysis under the method mentioned above. A new peak attributed to leucettazole A2 (**1b**) was observed when **1a** was treated with MeOH (Figures S18 and S19, Supplementary Materials), while a new peak attributed to leucettazole A (**1**) was observed when **1a** was treated with 0.02% TFA/H_2_O (Figures S22 and S23, Supplementary Materials).

### 3.6. UHPLC-QTOF (SIE) Analysis of Crude Extracts

An aliquot (1.0 mL) of crude EtOH extract of *Leucetta* sp. (CMB-01047) was concentrated in vacuo, reconstituted in 50% MeCN/H_2_O, and subjected to UHLPC-QTOF-MS/MS analysis with UHPLC conditions as mentioned previously. A UHPLC-QTOF analysis using SIE at *m/z* 453.1500 (peak width ± 0.05 amu) detected a species isomeric with leucettazole A2 (**1b**), but eluting at a different retention time (t_R_ 0.77 min versus 0.81 min, Figure S20, Supplementary Materials). This new species is consistent with the predicted natural product leucettazole B (**2**) (*m/z* 453.1516 [M + H]^+^, calculated for C_21_H_21_N_6_O_6_^+^, 453.1517). MS/MS analysis of **2** revealed diagnostic fragmentation consisting of the loss of OH (*m/z* 436.1249) (Figure S21, Supplementary Materials).

### 3.7. GNPS Analyses

MS/MS data were acquired by subjecting an aliquot of CMB-01047 *n*-butanol solubles (1 μL) to UHPLC-QTOF analysis. UHPLC conditions involved a 0.5-mL/min gradient elution from 10% CH_3_CN/H_2_O to 100% CH_3_CN over a period of 4.5 min, with constant 0.1% formic acid, through an Agilent SB-C_8_ 1.8-μm, 2.1 × 50 mm column. The source parameters were as follows: electrospray positive ionization; mass range of *m/z* 50–1700; scan rate, 10× per second; MS/MS scan rate, 3× per second; fixed collision energy, 40 eV; source gas temperature, 325 °C; gas flow, 10 L/min; and nebulizer, 20 psig. The scan source parameters were as follows: VCap 4000; fragmentor 100; skimmer 45; and octopole RF Peak 750. The acquired MS/MS data were converted from an Agilent MassHunter data file (.d) to the mzXML file format using the software MS-Convert [25]. Molecular networks were generated using the online Global Natural Products Social molecular networking web-platform (GNPS) (gnps.ucsd.edu). MS-Cluster with a precursor ion mass tolerance of 2.0 Da and an MS/MS fragment ion tolerance of 0.5 Da were selected to create consensus spectra [26]. A minimum cluster size of two, cosine score 0.65, and minimum number of fragments of six were selected for molecular networking. The spectral networks were imported into Cytoscape 3.5.1 [27] and visualized using force-directed layout where nodes represented parent masses and edge thickness corresponded to cosine score.

### 3.8. Antibacterial Assays

The bacterium to be tested was streaked onto a tryptic soy agar plate and was incubated at 37 °C for 24 h. One colony was then transferred to fresh tryptic soy broth (15 mL) and the cell density was adjusted to 10^4^–10^5^ colony-forming units (CFU)/mL. The compounds to be tested were dissolved in DMSO and diluted with H_2_O to give a 600 µM stock solution (20% DMSO), which was serially diluted with 20% DMSO to give concentrations from 600 µM to 0.2 µM in 20% DMSO. An aliquot (10 µL) of each dilution was transferred to a 96-well microtiter plate and freshly prepared microbial broth (190 µL) was added to each well to give final concentrations of 30–0.01 µM in 1% DMSO. The plates were incubated at 37 °C for 24 h, and the optical density of each well was measured spectrophotometrically at 600 nm using POLARstar Omega plate (BMG LABTECH, Offenburg, Germany). Each test compound was screened against the Gram-negative bacteria *Escherichia coli* ATCC 25922 and *Pseudomonas aeruginosa* ATCC 10145 and the Gram-positive bacteria *Staphylococcus aureus* ATCC 25923 and *Bacillus subtilis* ATCC 6633. Rifampicin was used as a positive control (40 µg/mL in 10% DMSO). For *Pseudomonas* *aeruginosa*, a mixture (1:1) of rifampicin and ampicillin (40 µg/mL in 10% DMSO) was used as a positive control. The IC_50_ value was calculated as the concentration of the compound or antibiotic required for 50% inhibition of the bacterial cells using Prism 7.0 (GraphPad Software Inc., La Jolla, CA). The antibacterial results are presented in Figure S31 (Supplementary Materials).

### 3.9. Antifungal Assay

The fungus *Candida albicans* ATCC 10231 was streaked onto a Sabouraud agar plate and was incubated at 37 °C for 48 h. One colony was then transferred to fresh Sabouraud broth (15 mL) and the cell density adjusted to 10^4^–10^5^ CFU/mL. Test compounds were dissolved in DMSO and diluted with H_2_O to give a 600 µM stock solution (20% DMSO), which was serially diluted with 20% DMSO to give concentrations from 600 µM to 0.2 µM in 20% DMSO. An aliquot (10 µL) of each dilution was transferred to a 96-well microtiter plate, and freshly prepared fungal broth (190 µL) was added to each well to give final concentrations of 30–0.01 µM in 1% DMSO. The plates were incubated at 37 °C for 24 h and the optical density of each well was measured spectrophotometrically at 600 nm using POLARstar Omega plate (BMG LABTECH, Offenburg, Germany). Amphotericin B was used as a positive control (30 µg/mL in 10% DMSO). Where relevant, IC_50_ values were calculated as the concentration of the compound or antifungal drug required for 50% inhibition of the fungal cells using Prism 7.0 (GraphPad Software Inc., La Jolla, CA). The antifungal results are presented in Figure S32 (Supplementary Materials).

### 3.10. Cytotoxicity Assays

Adherent human colorectal carcinoma (SW620) and human embryonic kidney (HEK293) cells were cultured in Roswell Park Memorial Institute (RPMI) medium 1640. All cells were cultured as adherent monolayers in flasks supplemented with 10% foetal bovine serum, l-glutamine (2 mM), penicillin (100 unit/mL), and streptomycin (100 μg/mL), in a humidified 37 °C incubator supplied with 5% CO_2_. Briefly, cells were harvested with trypsin and dispensed into 96-well microtiter assay plates at 3000 cells/well after which they were incubated for 18 h at 37 °C with 5% CO_2_ (to allow cells to attach as adherent monolayers). Test compounds were dissolved in 20% DMSO in phosphate-buffered saline (PBS) (*v*/*v*), and aliquots (10 μL) were applied to cells over a series of final concentrations ranging from 10 nM to 30 μM. After 48 h of incubation at 37 °C with 5% CO_2_, an aliquot (20 μL) of 3-(4,5-dimethylthiazol-2-yl)-2,5-diphenyltetrazolium bromide (MTT) in PBS (5 mg/mL) was added to each well (final concentration 0.5 mg/mL), and microtiter plates were incubated for a further 4 h at 37 °C with 5% CO_2_. After final incubation, the medium was aspirated, and precipitated formazan crystals dissolved in DMSO (100 μL/well). The absorbance of each well was measured at 580 nm with a PowerWave XS Microplate Reader from Bio-Tek Instruments Inc. (Vinooski, VT, USA). IC_50_ values were calculated using Prism 7.0 (GraphPad Software Inc., La Jolla, CA, USA), as the concentration of analyte required for 50% inhibition of cancer cell growth (compared to negative controls). Negative controls comprised 1% aqueous DMSO, while positive controls used vinblastine as the test sample. All experiments were performed in duplicate. The cytotoxicity results are presented in Figure S33 (Supplementary Materials).

## 4. Conclusions

Knowledge of leucettazoles A (**1**) and B (**2**) is an intriguing late addition to our understanding of the natural product scaffolds embodied within southern Australian marine sponges, and the proposed biosynthesis provides useful insights into a prospective biomimetic synthesis. Significantly, our discovery of the leucettazoles showcases three important aspects of modern natural product science. The first is the importance of valuing and exploring the potential of artifacts, such as leucettazoles A1 (**1a**) and B1 (**2a**), as a window into the world of otherwise reactive (and cryptic) natural products. The second is the remarkable capacity of modern UHPLC-QTOF methods, supported by techniques such as GNPS and SIE visualization, and MS/MS analysis, to give access to new and exciting chemical diversity, overlooked by earlier generations of researchers. The third is the potential relationship between natural products, artifacts, and biological activity. The fact that the leucettazole ethanolysis adducts **1a** and **2a** did not exhibit cytotoxic properties against a panel of bacterial, fungal, and mammalian cells is perhaps not surprising, and should not discount the possibility that natural products **1** and **2** are bioactive. Much as is the case with Michael acceptors and other bioactive molecules that form covalent bonds with biological targets, it is possible that **1** and **2** are bioactive due to their innate solvolytic reactivity,. If correct, the solvolysis products **1a** and **2a** could be viewed as inactivated adducts, much as Michael adducts deactivate Michael acceptors. While the ecological purpose and pharmacological potential of the leucettazoles remains a mystery, we contend that this scaffold is deserving of further investigation. That said, given their rarity in nature, and inherent chemical reactivity, further exploration will likely require the input of synthetic and medicinal chemists.

## Figures and Tables

**Table 1:** One-dimensional (1D) and two-dimensional (2D) NMR (dimethyl sulfoxide, DMSO-*d*_6_; 600 MHz) data for leucettazoles A1 (**1a**) (as trifluoroacetate (TFA) salts).

Position	*δ*_C_	*δ*_H_, mult. (*J* in Hz)	COSY	*g*HMBC	ROESY
2	166.7, C				
2-NH_2_		8.21, br s			3-NH
		8.00, br s			3-NH
3-NH		9.16, s		2, 4, 5, 2′	6a, 12, 4-OCH_2a_, 2-NH_2_
4	96.6, C				
4-OCH_2_CH_3_	59.0, CH_2_	a. 3.45, dq (8.8, 7.0)	4-OCH_b_CH_3_	4, 4-OCH_2_CH_3_	3, 4-OCH_b_, 4-OCH_2_CH_3_
		b. 3.31, dq (8.8, 7.0)	4-OCH_a_CH_3_	4, 4-OCH_2_CH_3_	4-OCH_a_, 4-OCH_2_CH_3_
4-OCH_2_CH_3_	15.1, CH_3_	1.16, dd (7.0, 7.0)	4-OCH_2_	4-OCH_2_	4-OCH_2_
5	178.3, C				
6	43.4, CH_2_	a. 3.03, d (13.1)		5, 7, 8, 12	3-NH, 12
		b. 2.81, d (13.1)		4, 5, 7, 8, 12	8
7	124.3, C				
8	112.3, CH	5.99, d (2.0)	12	6, 9, 10, 12	6b, 12′, 12
9	144.8, C				
10	136.2, C				
10-OH		8.68, s		9, 10, 11	
11	146.1, C				
11-OH		8.87 ^a^		10, 12	
12	114.6, CH	6.57, d (2.0)	8	6, 8, 10, 11	6a, 8, 3-NH
2′	148.5, C				
3′-NH		11.82, s			
4′	127.0, C				
5′	112.9, CH	7.07, s		2′, 4′	6′a/b, 12′
6′	29.1, CH_2_	a. 3.82, d (16.7)		4′, 5′, 7′, 8′, 12′	5′, 8′, 12′
		b. 3.72, d (16.7)		4′, 5′, 7′, 8′, 12′	5′, 8′, 12′
7′	130.0, C				
8′	111.4, CH	6.51, d (2.0)	12′	6′, 9′, 10′, 12′	6′a/b, 12′, 9′-OH
9′	146.6, C				
9′-OH		9.29, s		8′, 9′, 10′	8′
10′	134.1, C				
10′-OH		8.86 ^a^		9′	
11′	145.8, C				
12′	108.9, CH	5.46, d (2.0)	8′	6′, 8′, 10′, 11′	8, 5′, 6′a/b, 8′

^a^ Resonances with the same superscript within a column are interchangeable.

**Table 2:** 1D and 2D NMR (DMSO-*d*_6_, 600 MHz) data for leucettazole B1 (**2a**) (as a TFA salt).

Position	*δ*_C_	*δ*_H_, mult. (*J* in Hz)	COSY	*g*HMBC
2	166.5, C			
2-NH_2_		a. 8.23, s		
		b. 8.02, s		
3-NH		9.20, s		2, 4, 5
4	95.9, C			
4-OCH_2_CH_3_	58.4, CH_2_	a. 3.45, dq (8.8, 7.0)	4-OCH_b_CH_3_	4-OCH_2_CH_3_
		b. 3.28, dq (8.8, 7.0)	4-OCH_a_CH_3_	
4-OCH_2_CH_3_	14.6, CH_3_	1.15, dd (7.0, 7.0)	4-OCH_2_	4-OCH_2_
5	177.5, C			
6	42.7, CH_2_	a. 3.07, d (13.2)		4, 5, 7, 8, 12
		b. 2.75, d (13.2)		4, 5, 7, 8, 12
7	123.9, C			
8	111.5, CH	5.92, d (2.0)	12	6, 9, 10, 12
9	143.1, C			
10	135.7 ^a^, C			
10-OH		8.52, s		9, 11
11	145.9, C			
11-OH		8.91, s		10, 11, 12
12	112.8, CH	6.53, d (2.0)	8	6, 8, 11
2′	148.0, C			
3′-NH		12.1, br s		
4′	126.9, C			
5′	112.3, CH	6.97, s		2′, 4′
6′	29.0, CH_2_	a. 3.86, d (16.2)		4′, 5′, 7′, 8′
		b. 3.63, d (16.2)		4′, 5′, 7′, 8′, 12′
7′	134.6, C			
8′	110.7, CH	6.52, d (2.0)	12′	6′, 9′, 10′, 12′
9′	150.3, C			
9′-OH		9.37, s		8′, 9′, 10′
10′	135.6 ^a^, C			
10′-OCH_3_	59.9, CH_3_	3.71, s		10′
11′	149.9, C			
12′	107.1, CH	5.43, d (2.0)	8′	6′, 8′, 10′, 11′

^a^ Resonances with the same superscript within a column are interchangeable.

**Table 3:** 1D and 2D NMR (DMSO-d6, 600 MHz) data for leucettazine A (**3**) (as a TFA salt).

Position	*δ*_C_	*δ*_H_, mult. (*J* in Hz)	COSY	*g*HMBC
1-NMe	31.8, CH_3_	3.37, s		2, 5
2	146.2, C			
2-NH_2_		6.54, br s		
3-NH		12.06, s		
4	125.9, C			
5	113.7, CH	6.61, s		2, 4
6	29.4, CH_2_	3.58, s		4, 5, 7, 8, 12
7	128.1, C			
8	119.1, CH	6.47, dd (8.0, 1.5)	9,12	6, 10, 12
9	115.5, CH	6.66, d (8.0)	8	7, 11
10	144.0, C			
10-OH		7.43, s		
11	145.2, C			
11-OH		8.82, s		10, 11, 12
12	115.9, CH	6.59, d (1.5)	8	6, 8, 10

**Table 4:** ^1^H and ^13^C NMR (DMSO-*d*_6_, 600 MHz) data for leucettazole A (**1**) (as a TFA salt).

Position	*δ*_C_	*δ*_H_, mult. (*J* in Hz)	Position	*δ*_C_	*δ*_H_, mult. (*J* in Hz)
2	165.9, C		12	114.6, CH	6.55, d (2.0)
2-NH_2_		a. 7.94, br s	2′	148.5, C	
		b. 7.83, br s	3′-NH		11.73, br s
3-NH		9.17, s	4′	126.8, C	
4	92.1, C		5′	112.9, CH	7.05, s
4-OH		6.86, s	6′	29.1, CH_2_	a. 3.81, d (16.7)
5	180.8, C				b. 3.72, d (16.7)
6	44.1, CH_2_	a. 2.98, d (13.1)	7′	130.0, C	
		b. 2.80, d (13.1)	8′	111.3, CH	6.51, d (2.0)
7	125.1, C		9′	146.5, C	
8	112.3, CH	5.98, d (2.0)	9′-OH		9.29, s
9	144.7, C		10′	134.1, C	
10	136.1, C		10′-OH		8.85
10-OH		8.67, s	11′	145.9, C	
11	146.0, C		12′	109.0, CH	5.48, d (2.0)
11-OH		8.88			

## Supplementary Materials

The following are available online at http://www.mdpi.com/1660-3397/17/2/106/s1, annotated 1D NMR and selected 2D NMR spectra, MS/MS spectra, and bioassay data (pdf).
