# Supplementary material for: Solvolysis Artifacts: Leucettazoles as Cryptic Macrocyclic Alkaloid Dimers from a Southern Australian Marine Sponge, Leucetta sp
Source: Mar Drugs. 2019 Feb 9;17(2):106. doi: 10.3390/md17020106 (PMC6410009; doi:10.3390/md17020106)
Supplement: Supplementary file 1 [file marinedrugs-17-00106-s001.pdf]

Supporting Information

Solvolysis Artifacts: Leucettazoles as Cryptic Macrocyclic Alkaloid Dimers from a Southern Australian Marine Sponge, *Leucetta* sp.

Pritesh Prasad, Angela A. Salim, Shamsunnahar Khushi, Zeinab G. Khalil, Michelle Quezada and Robert J. Capon*

Institute for Molecular Bioscience, The University of Queensland, St Lucia, QLD 4072, Australia; p.prasad@imb.uq.edu.au (P.P.), a.salim@uq.edu.au (A.A.S.), s.khushi@imb.uq.edu.au (S.K.), z.khalil@imb.uq.edu.au (Z.G.K.), michelle.quezada@newcastle.edu.au (M.Q.)

* Correspondence: r.capon@uq.edu.au (R.J.C.); Tel.: +61 7 3346 2979

***Corresponding author**

Tel.: +61 7 3346 2979. Fax: +61 7 3346 2090. E-mail: r.capon@uq.edu.au

Table of Contents

Figure S1. ^{1}H NMR (DMSO-d_6, 600 MHz) spectrum of leucettazole A1 (**1a**) 4

Figure S2. ^{13}C NMR (DMSO-d_6, 150 MHz) spectrum of leucettazole A1 (**1a**) 4

Figure S3. HSQC NMR (DMSO-d_6, 600 MHz) spectrum of leucettazole A1 (**1a**) 5

Figure S4. HMBC NMR (DMSO-d_6, 600 MHz) spectrum of leucettazole A1 (**1a**) 5

Figure S5. Expanded HMBC NMR (DMSO-d_6, 600 MHz) spectrum of leucettazole A1 (**1a**)6

Figure S6. ROESY NMR (DMSO-d_6, 600 MHz) spectrum of leucettazole A1 (**1a**) 6

Figure S7. ^{1}H -^{15}N HSQC (DMSO-d_6, 600 MHz) spectrum of leucettazole A1 (**1a**) 7

Figure S8. ^{1}H NMR (DMSO-d_6, 600 MHz) spectrum of leucettazole B1 (**2a**) 8

Figure S9. ^{13}C NMR (DMSO-d_6, 150 MHz) spectrum of leucettazole B1 (**2a**) 8

Figure S10.^{1}H NMR (DMSO-d_6, 600 MHz) spectrum of leucettazine A (**3**) 9

Figure S11. ^{13}C NMR (DMSO-d_6, 150 MHz) spectrum of leucettazine A (**3**) 9

Figure S12. Comparison of ^{1}H NMR (DMSO-d_6, 600 MHz) expanded spectra: (a) leucettazole A1 (**1a**), (b) mixture of leucettazole A (**1**) and leucettazine A (**3**), and (c) leucettazine A (**3**) 10

Figure S13. Comparison of ^{1}H NMR (DMSO-d_6, 600 MHz) expanded spectra: (a) leucettazole A1 (**1a**), (b) mixture of leucettazole A (**1**) and leucettazine A (**3**), and (c) leucettazine A (**3**) 10

Figure S14. Comparison of ^{1}H NMR (DMSO-d_6, 600 MHz) expanded spectra: (a) leucettazole A1 (**1a**), (b) mixture of leucettazole A (**1**) and leucettazine A (**3**), and (c) leucettazine A (**3**) 11

Figure S15. ^{13}C NMR (DMSO-d_6, 150 MHz) spectrum with carbons numbers in black for leucettazole A (**1a**) and carbon numbers in red for leucettazine A (**3**) 11

Figure S16. UHPLC-QTOF analysis of crude EtOH extract treated with MeOH at 60 °C. ..12

Figure S17. UHPLC-QTOF-MS/MS analysis of crude EtOH extract treated with MeOH at 60 °C 12

Figure S18. UHPLC-QTOF analysis of leucettazole A1 (**1a**) treated with MeOH at 40 °C. .13

Figure S19. UHPLC-QTOF-MS/MS analysis of leucettazole A1 (**1a**) treated with MeOH at 40 ° 13

Figure S20. UHPLC-QTOF analysis of crude EtOH treated with aqueous MeCN. 14

Figure S21. UHPLC-QTOF-MS/MS analysis of crude EtOH extract treated with aqueous MeCN 14

Figure S22. UHPLC-QTOF analysis of leucettazole A1 (**1a**) treated with 0.02% TFA/H_2O at 60 °C. 15

Figure S23. UHPLC-QTOF-MS/MS analysis of leucettazole A1 (**1a**) treated with 0.02% TFA/H_2O at 60 °C 15

Figure S24. UHPLC-QTOF-MS/MS analysis of CMB-01047 *n*-BuOH solubles (top) +ESI TIC scan at t_R 0.556 min representing leucettazole A (**1**) (*m/z* 439.1362); (bottom) MS/MS fragmentation for **1** (loss of OH resulting in *m/z* 422.1089). 16

Figure S25. UHPLC-QTOF-MS/MS analysis of CMB-01047 *n*-BuOH solubles (top) +ESI TIC scan at t_R 0.757 min representing leucettazole B (**2**) (*m/z* 453.1516); (bottom) MS/MS fragmentation for **2** (loss of OH resulting in *m/z* 436.1241).....16

Figure S26. UHPLC-QTOF-MS/MS analysis of CMB-01047 *n*-BuOH solubles (top) +ESI TIC scan at t_R 0.930 min representing leucettazole A1 (**1a**) (*m/z* 467.1706); (bottom) MS/MS fragmentation for **1a** (loss of OEt resulting in *m/z* 421.1272).....17

Figure S27. UHPLC-QTOF-MS/MS analysis of CMB-01047 *n*-BuOH solubles (top) +ESI TIC scan at t_R 1.064 min representing leucettazole B1 (**2a**) (*m/z* 481.1837); (bottom) MS/MS fragmentation for **2a** (loss of OEt resulting in *m/z* 435.1398).....17

Figure S28. UHPLC-QTOF-MS/MS analysis of CMB-01047 *n*-BuOH solubles (top) +ESI TIC scan at t_R 1.216 min representing **i** (*m/z* 435.1765) (calcd for $C_{22}H_{23}N_6O_4^+$, 435.1775); (bottom) MS/MS fragmentation for **i** (loss of OEt resulting in *m/z* 389.1349). 18

Figure S29. UHPLC-QTOF-MS/MS analysis of CMB-01047 *n*-BuOH solubles (top) +ESI TIC scan at t_R 1.357 min representing **ii** (*m/z* 495.1952) (calcd for $C_{24}H_{27}N_6O_6^+$, 495.1987); (bottom) MS/MS fragmentation for **ii** (loss of butyl ether resulting in *m/z* 421.1235). 18

Figure S30. UHPLC-QTOF-MS/MS analysis of CMB-01047 *n*-BuOH solubles (top) +ESI TIC scan at t_R 0.846 min representing **iii** (*m/z* 545.1464) (calcd for $C_{23}H_{25}N_6O_8S^+$, 545.1449); (bottom) MS/MS fragmentation for **iii** (loss of EtOH resulting in *m/z* 499.1048). 19

Figure S31. Antibacterial assay results for leucettazole A1 (**1a**) and leucettazole B1 (**2a**) ...20

Figure S32. Antifungal assay results for leucettazole A1 (**1a**) and leucettazole B1 (**2a**).......20

Figure S33. Cytotoxicity assay of leucettazole A1 (**1a**) and leucettazole B1 (**2a**) against (a) HEK293 (human embryonic kidney cell line) and (b) SW620 (human colon cancer cell line) 20

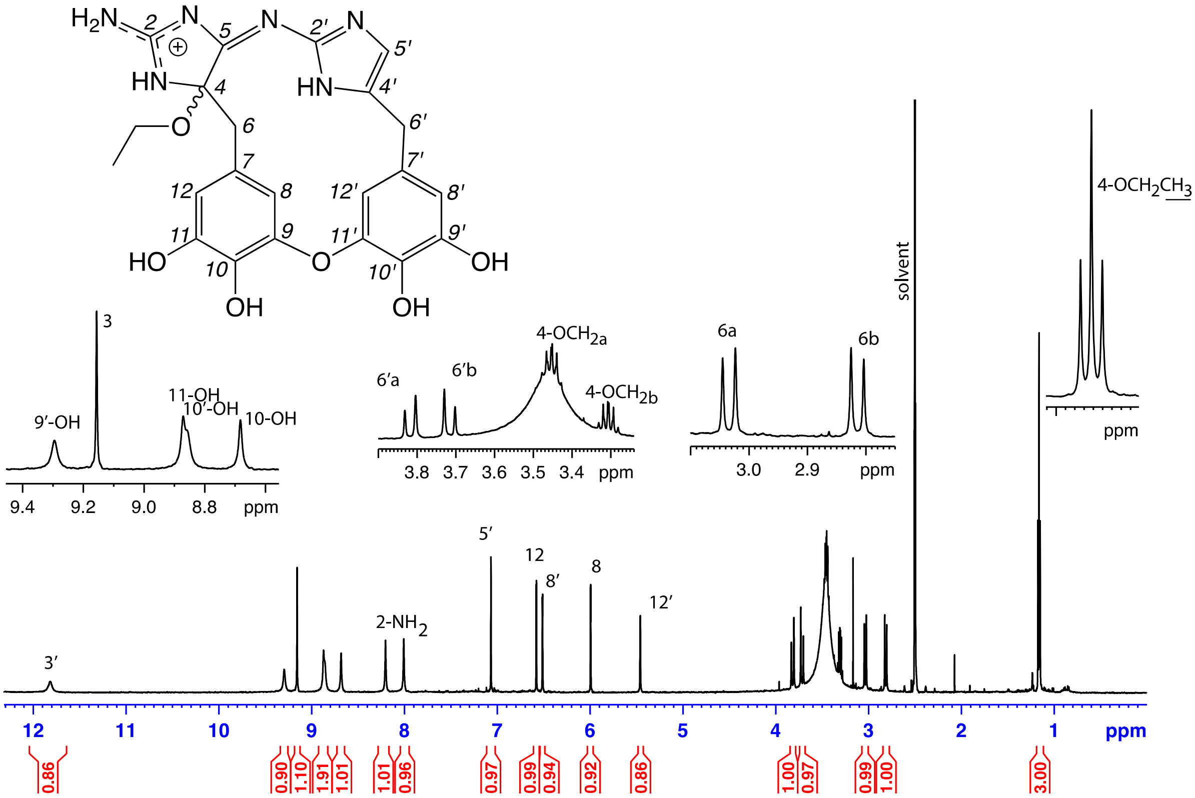

Figure S1. ^{1}H NMR (DMSO-d_6, 600 MHz) spectrum of leucettazole A1 (**1a**)

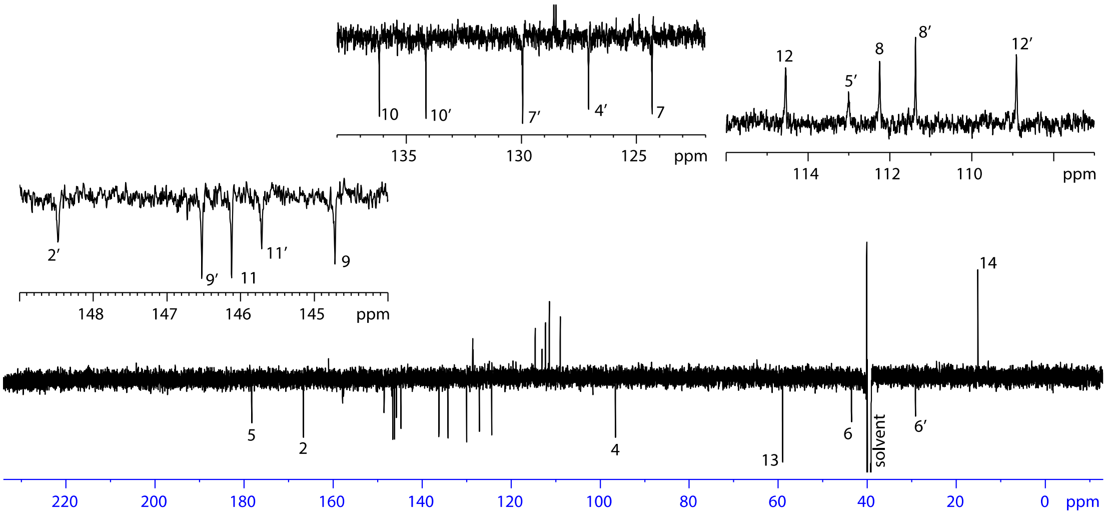

Figure S2. ^{13}C NMR (DMSO-d_6, 150 MHz) spectrum of leucettazole A1 (**1a**)

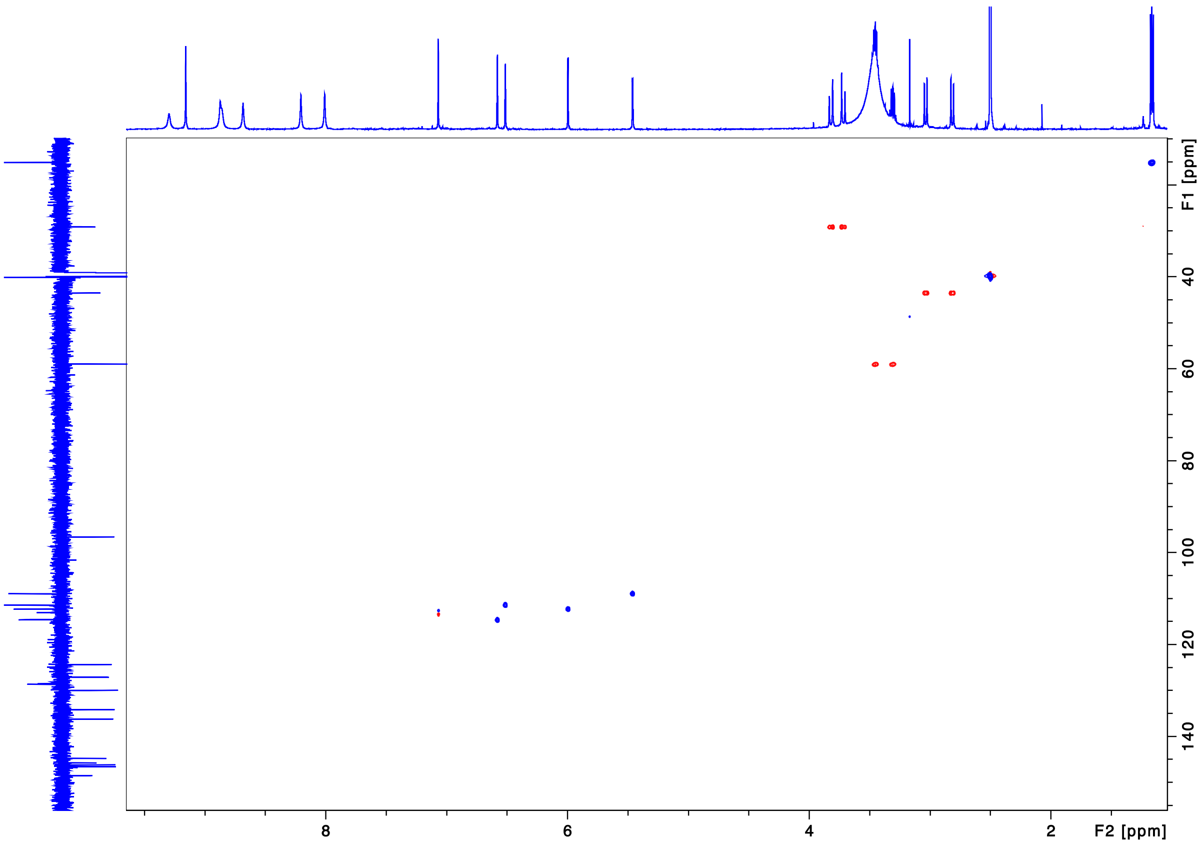

Figure S3. HSQC NMR (DMSO-d_6, 600 MHz) spectrum of leucettazole A1 (**1a**)

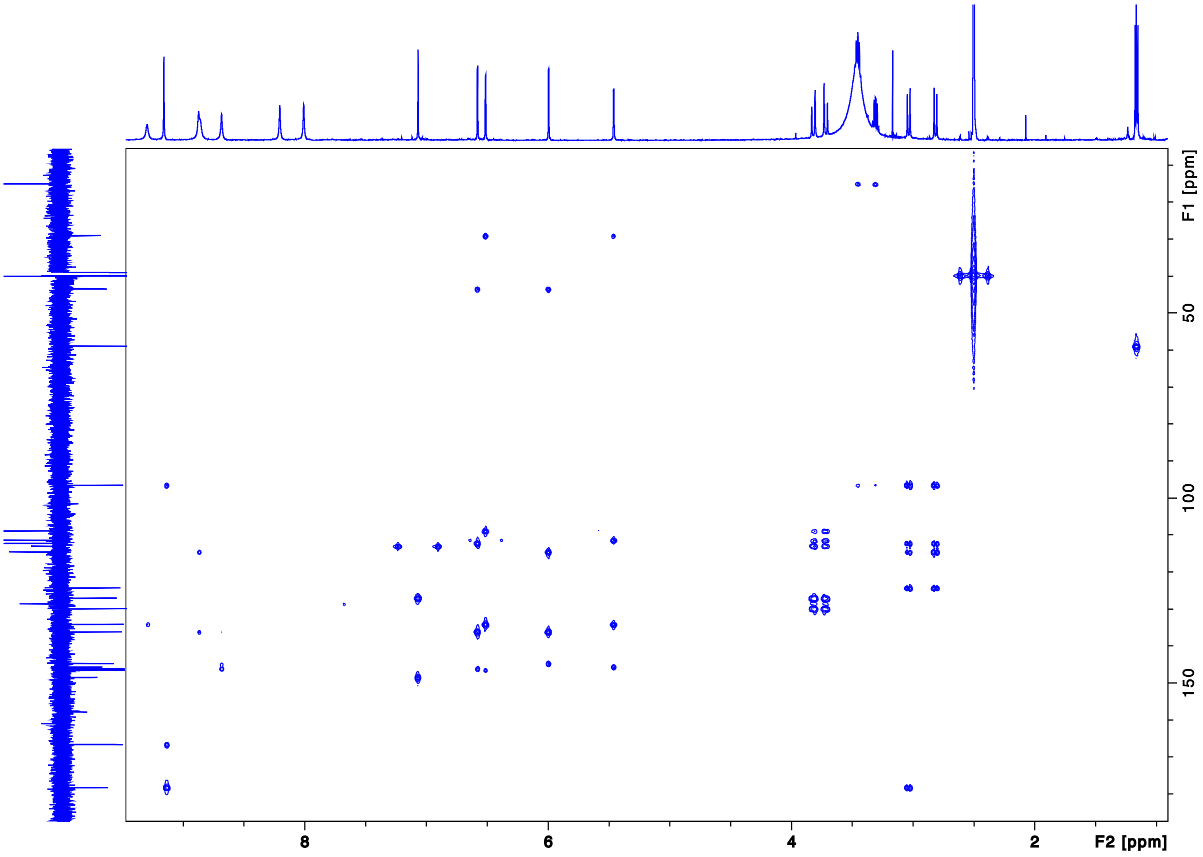

Figure S4. HMBC NMR (DMSO-d_6, 600 MHz) spectrum of leucettazole A1 (**1a**)

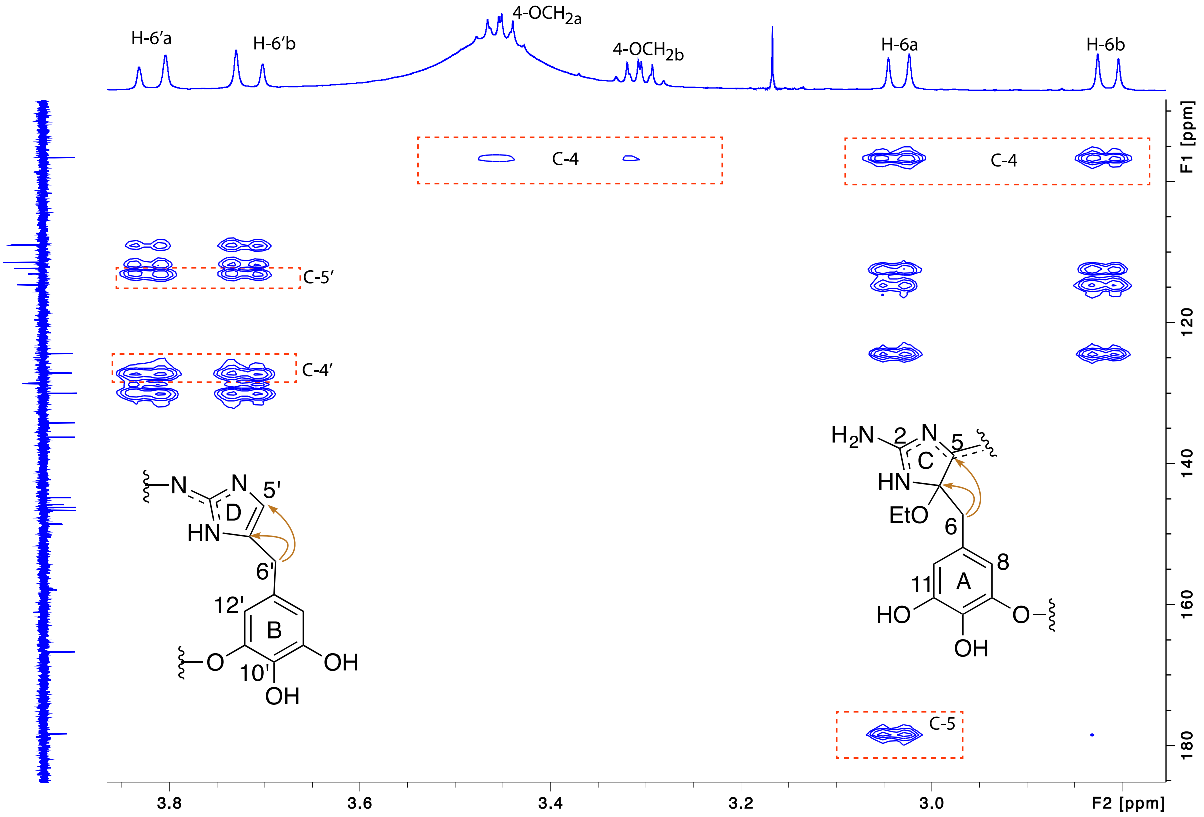

Figure S5. Expanded HMBC NMR (DMSO-d_6, 600 MHz) spectrum of leucettazole A1 (**1a**)

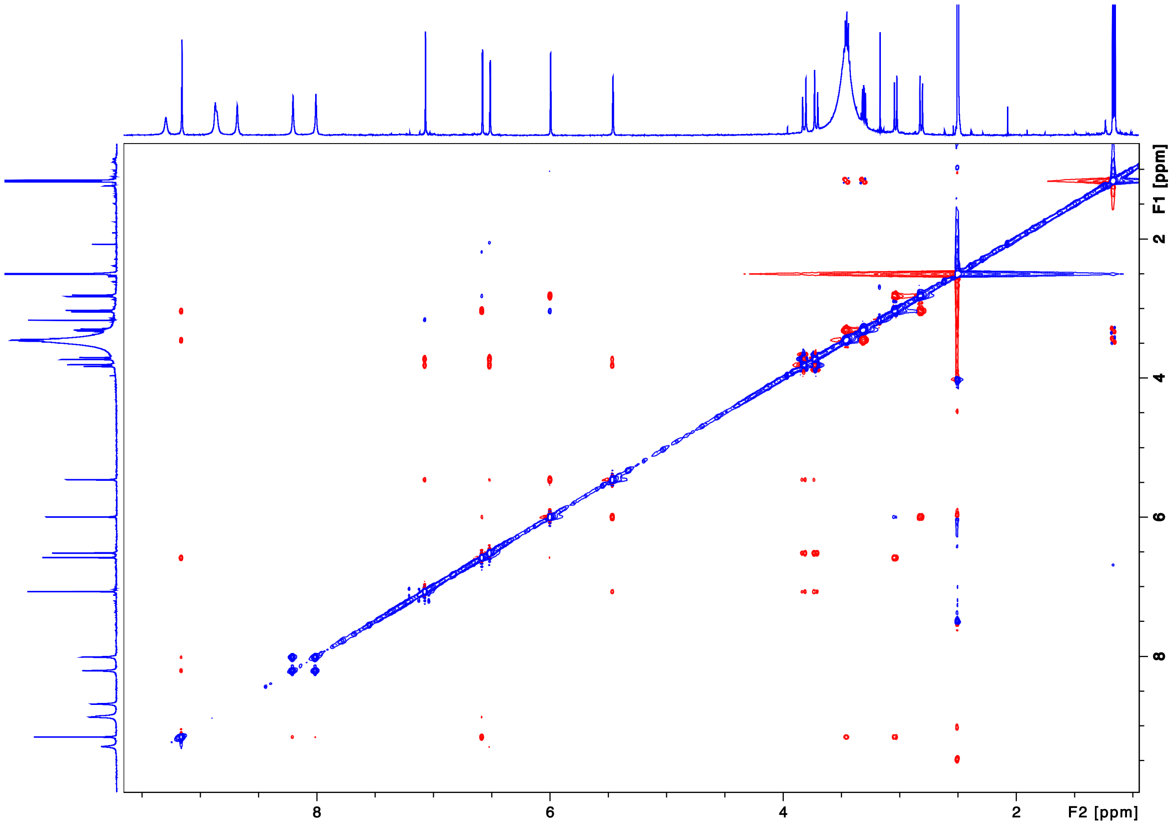

Figure S6. ROESY NMR (DMSO-d_6, 600 MHz) spectrum of leucettazole A1 (**1a**)

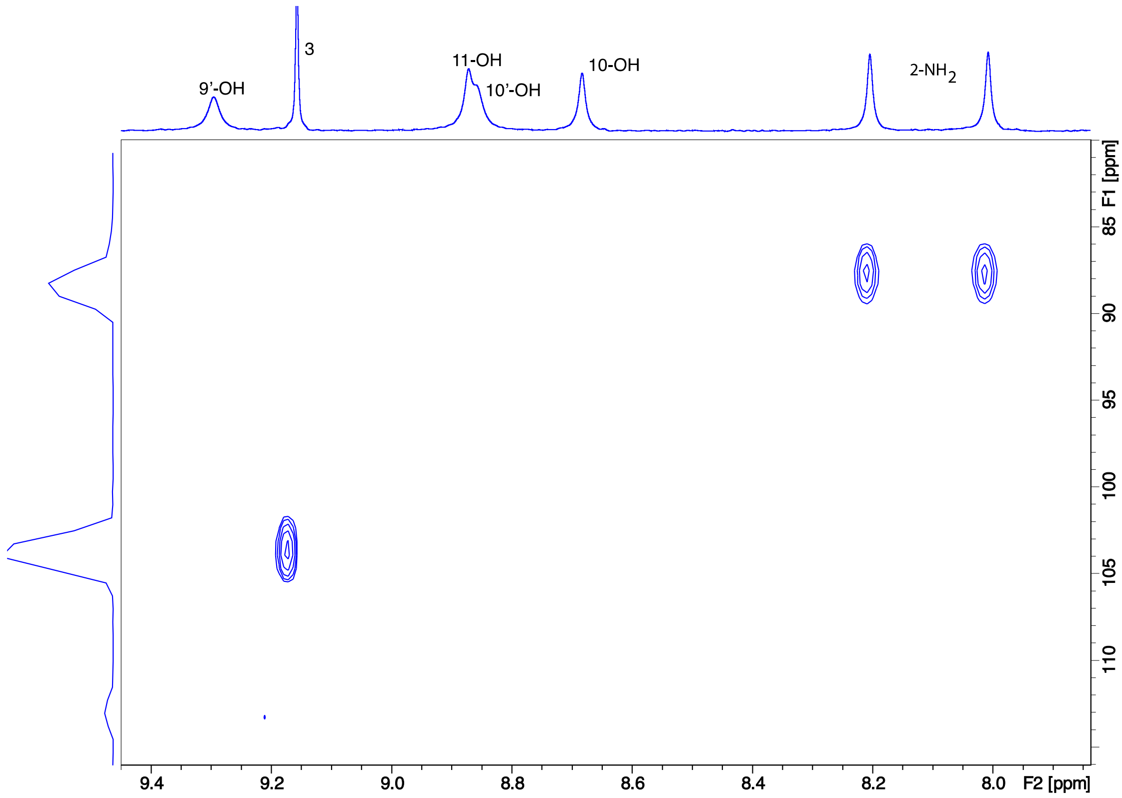

Figure S7. ^{1}H -^{15}N HSQC (DMSO-d_6, 600 MHz) spectrum of leucettazole A1 (**1a**)

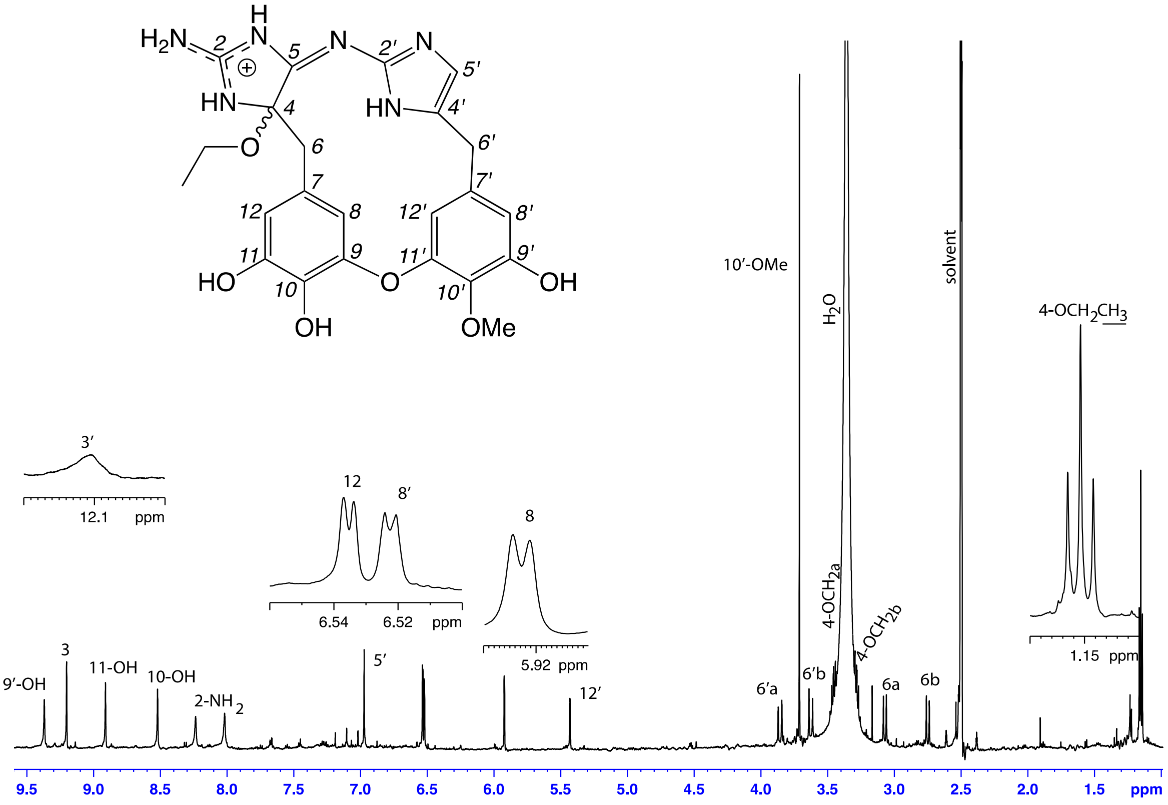

Figure S8. ^{1}H NMR (DMSO-d_6, 600 MHz) spectrum of leucettazole B1 (**2a**)

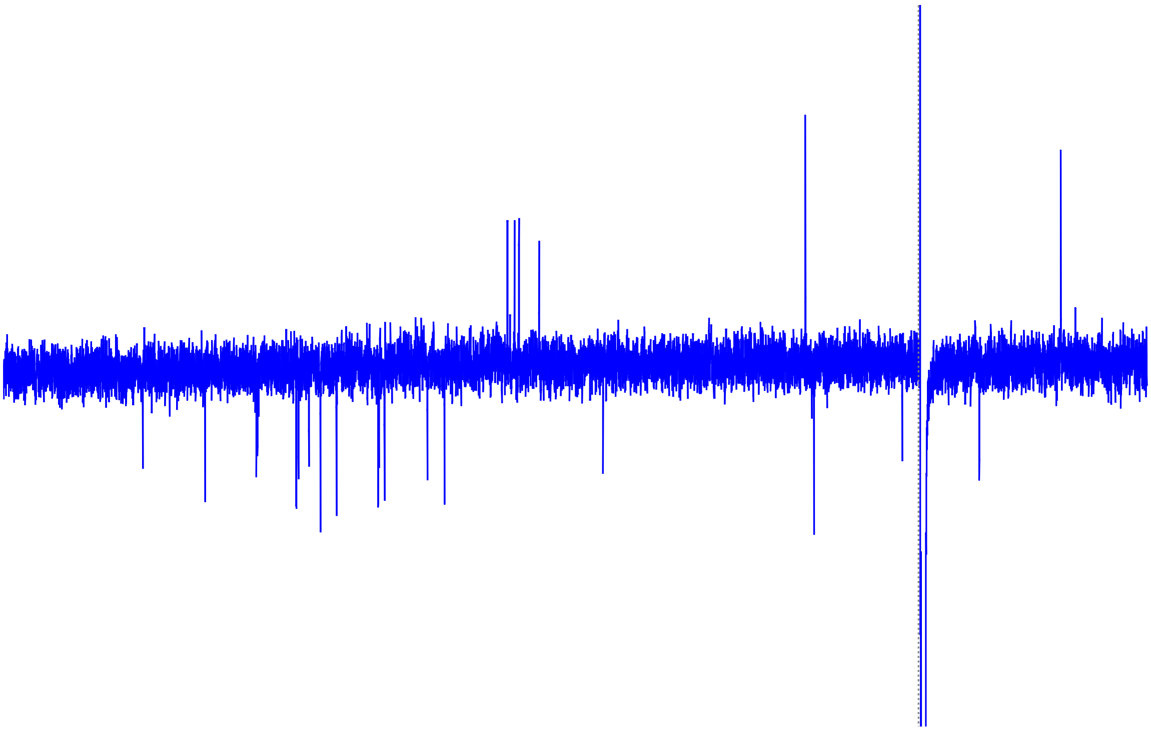

Figure S9. ^{13}C NMR (DMSO-d_6, 150 MHz) spectrum of leucettazole B1 (**2a**)

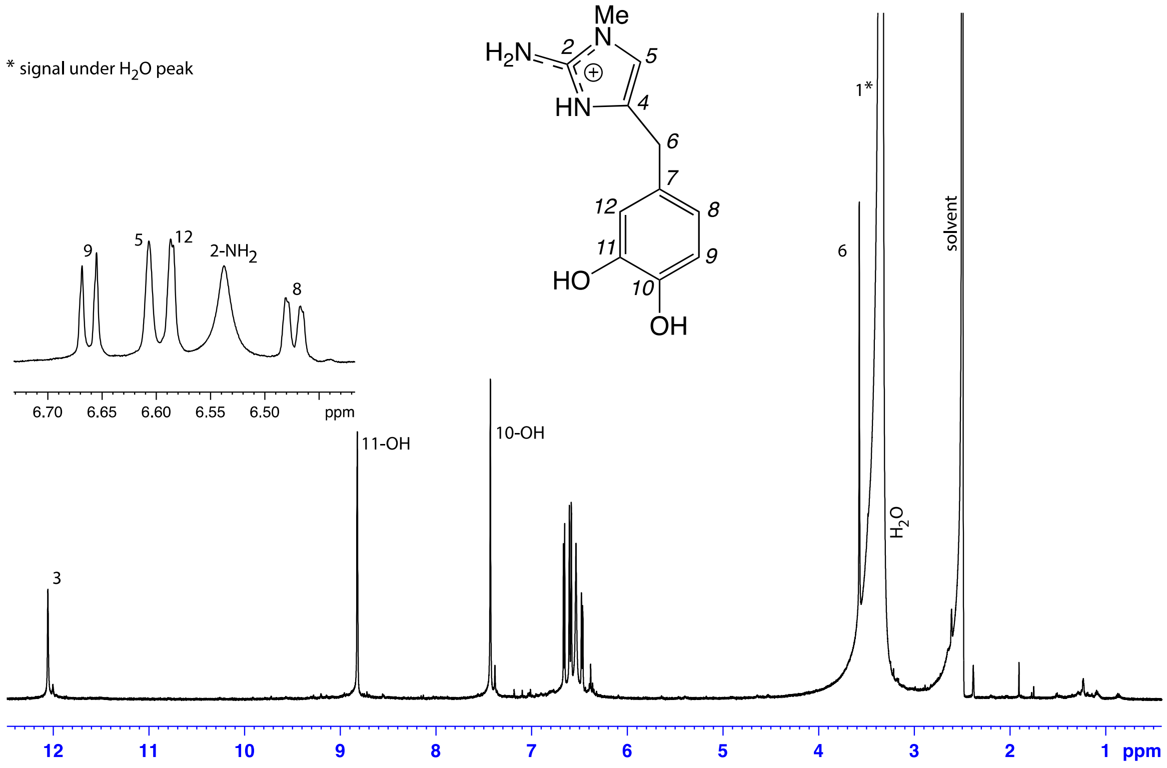

Figure S10.^{1}H NMR (DMSO-d_6, 600 MHz) spectrum of leucettazine A (**3**)

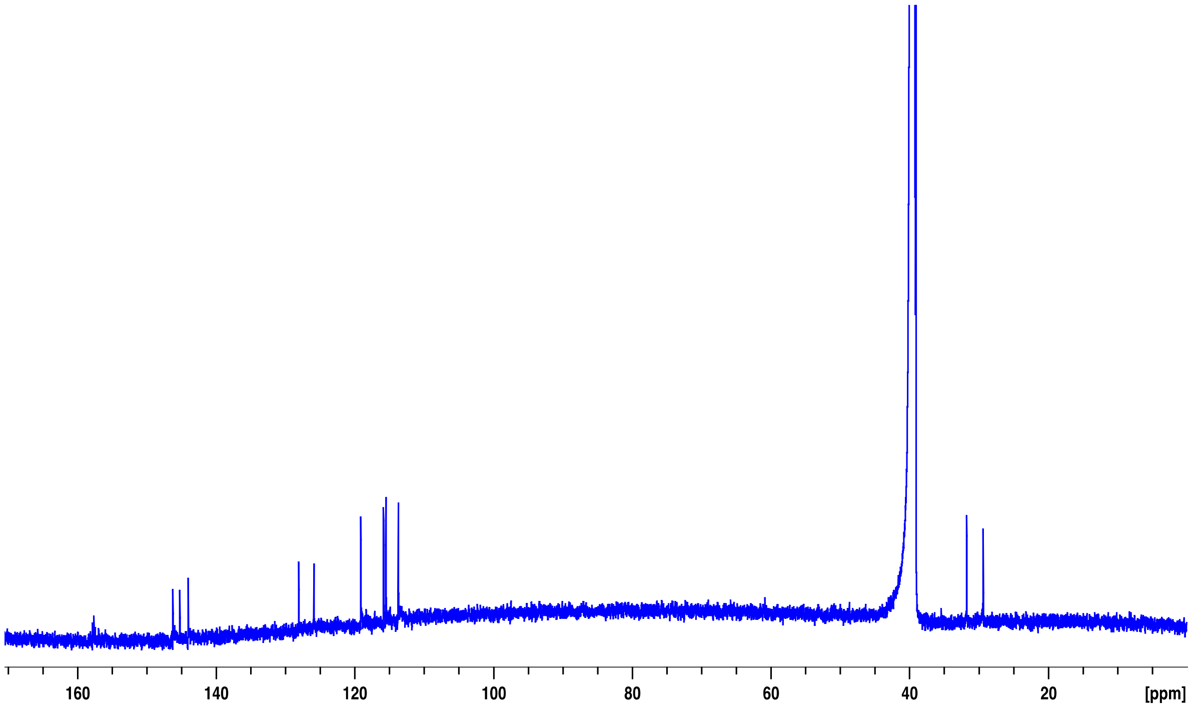

Figure S11. ^{13}C NMR (DMSO-d_6, 150 MHz) spectrum of leucettazine A (**3**)

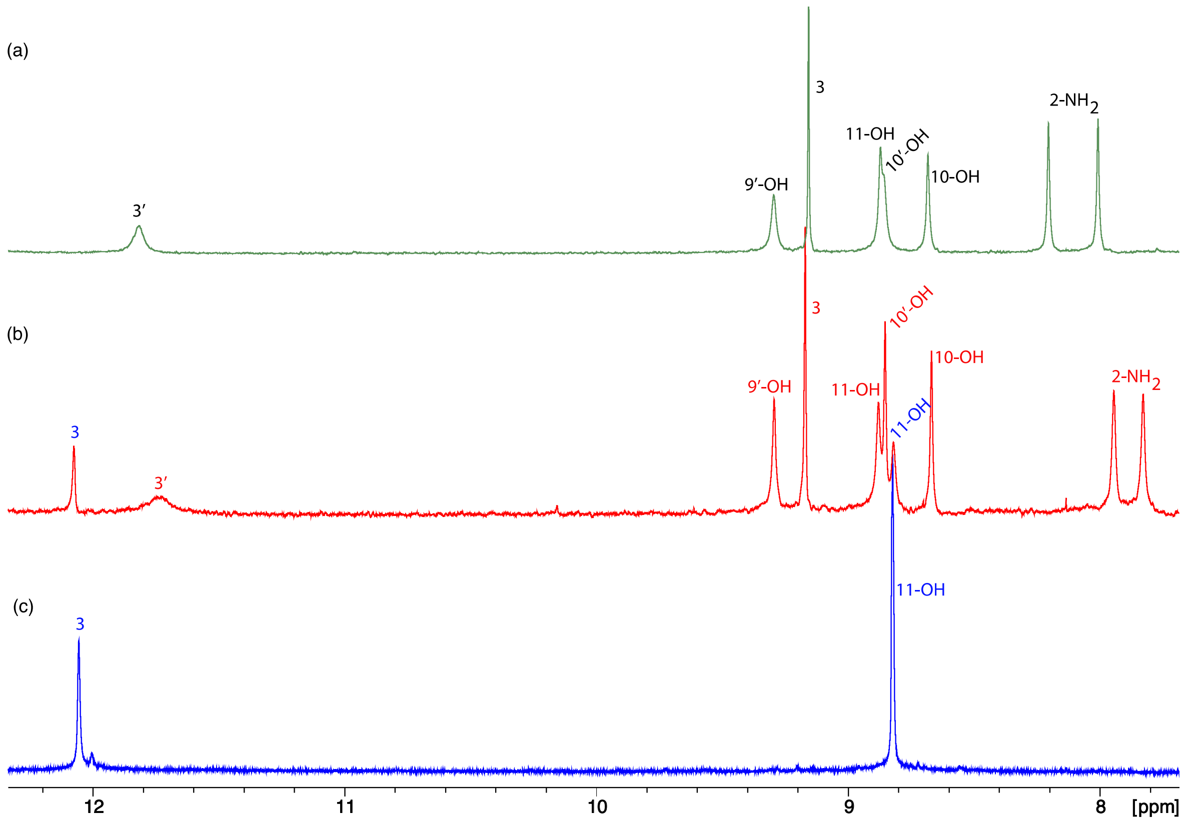

Figure S12. Comparison of ^{1}H NMR (DMSO-d_6, 600 MHz) expanded spectra: (a) leucettazole A1 (**1a**), (b) mixture of leucettazole A (**1**) and leucettazine A (**3**), and (c) leucettazine A (**3**)

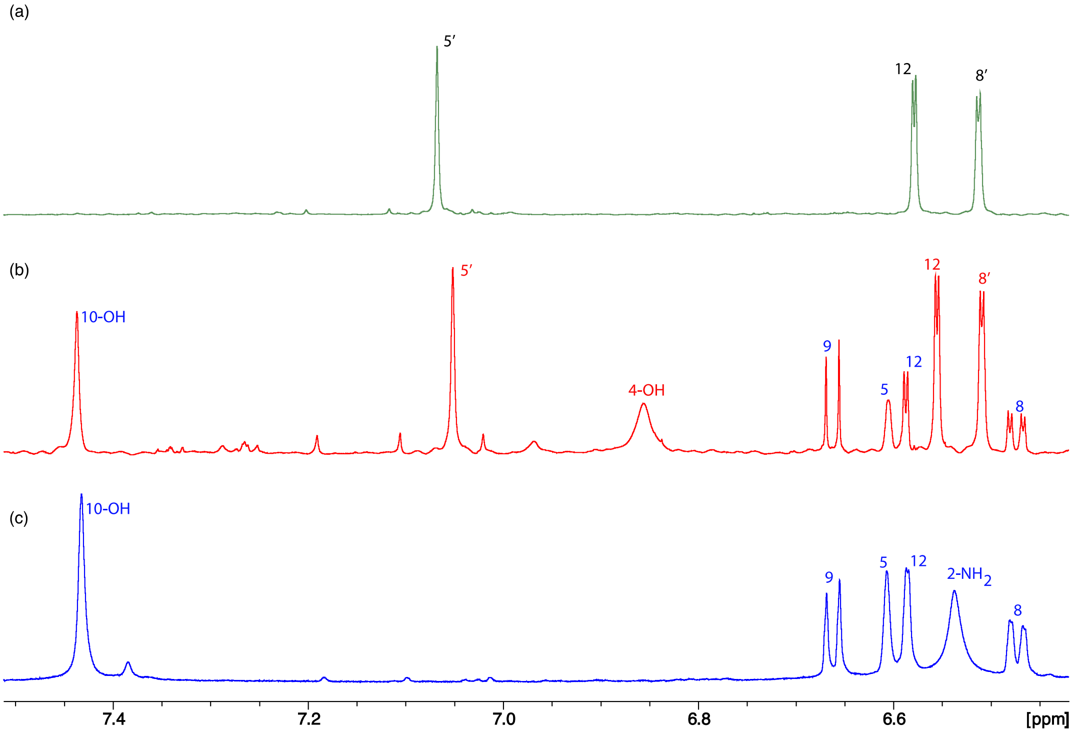

Figure S13. Comparison of ^{1}H NMR (DMSO-d_6, 600 MHz) expanded spectra: (a) leucettazole A1 (**1a**), (b) mixture of leucettazole A (**1**) and leucettazine A (**3**), and (c) leucettazine A (**3**)

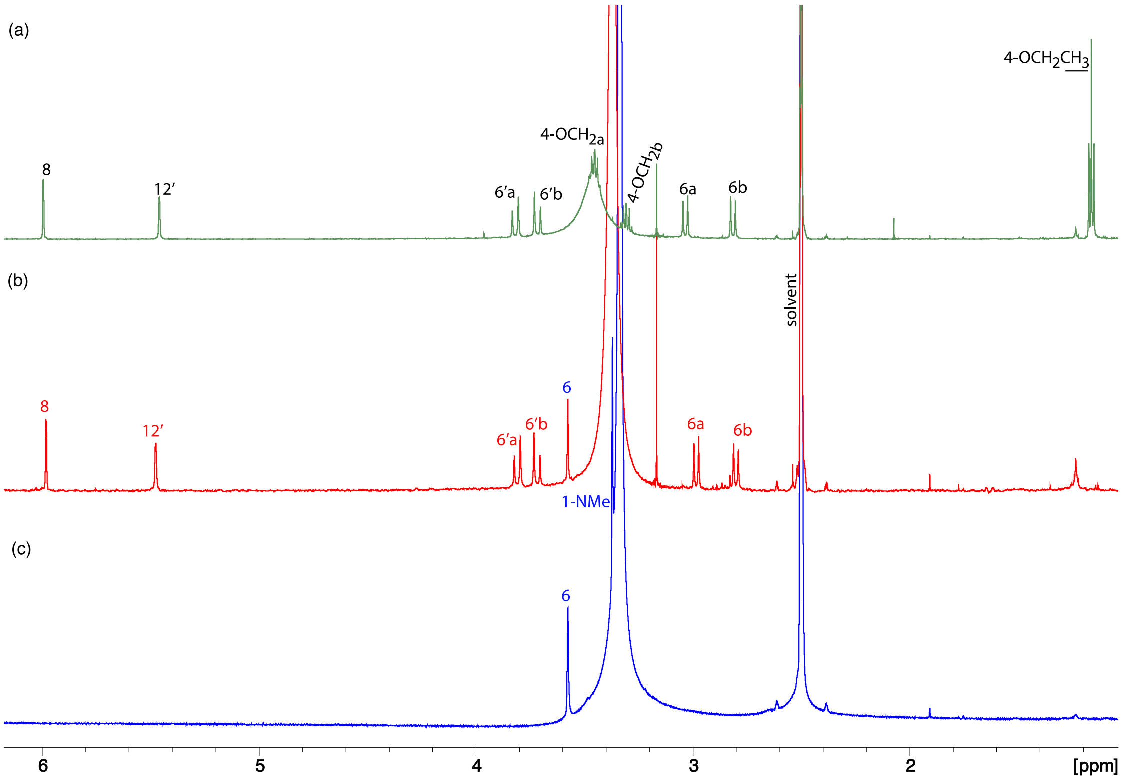

Figure S14. Comparison of ^{1}H NMR (DMSO-d_6, 600 MHz) expanded spectra: (a) leucettazole A1 (**1a**), (b) mixture of leucettazole A (**1**) and leucettazine A (**3**), and (c) leucettazine A (**3**)

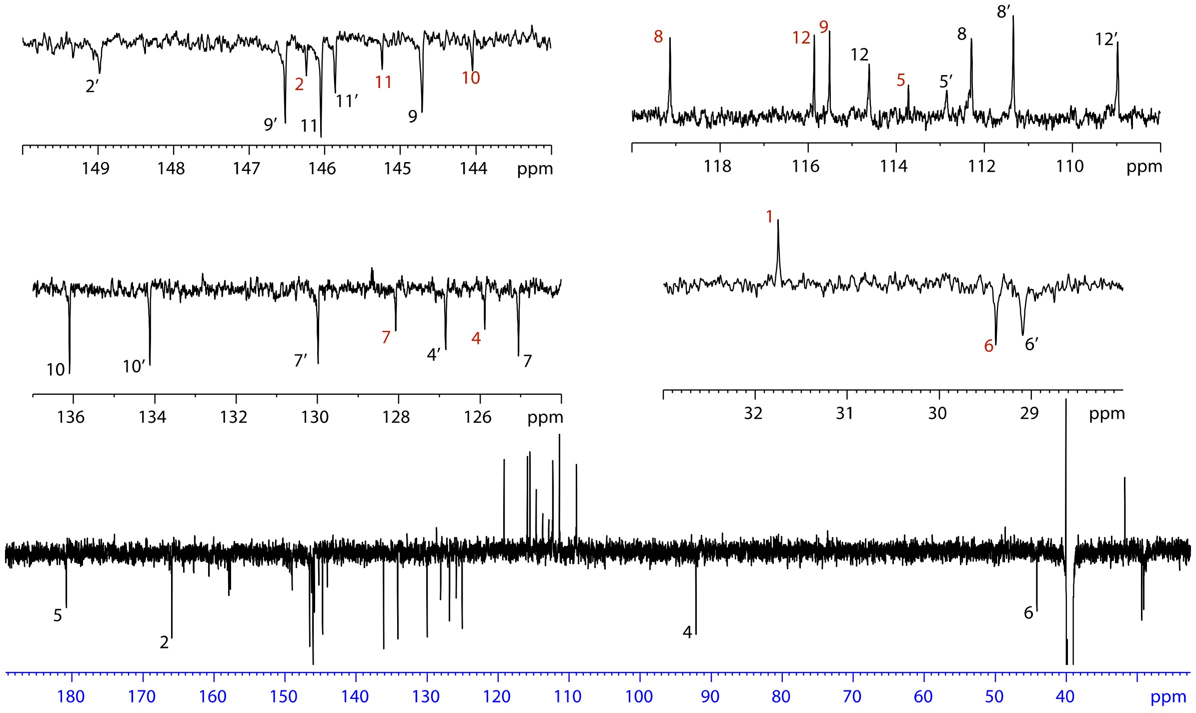

Figure S15. ^{13}C NMR (DMSO-d_6, 150 MHz) spectrum with carbons numbers in black for leucettazole A (**1a**) and carbon numbers in red for leucettazine A (**3**)

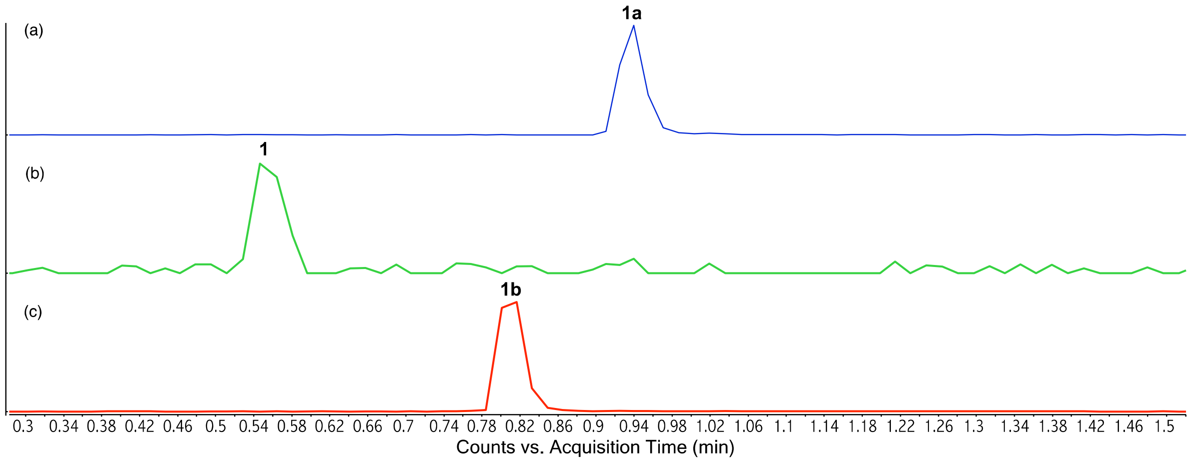

Figure S16. UHPLC-QTOF analysis of crude EtOH extract treated with MeOH at 60 °C. Single ion extraction (SIE) chromatograms for (a) *m/z* 467.17 (leucettazole A1, **1a**); (b) *m/z* 439.14 (leucettazole A, **1**); (c) *m/z* 453.15 (leucettazole A2, **1b**)

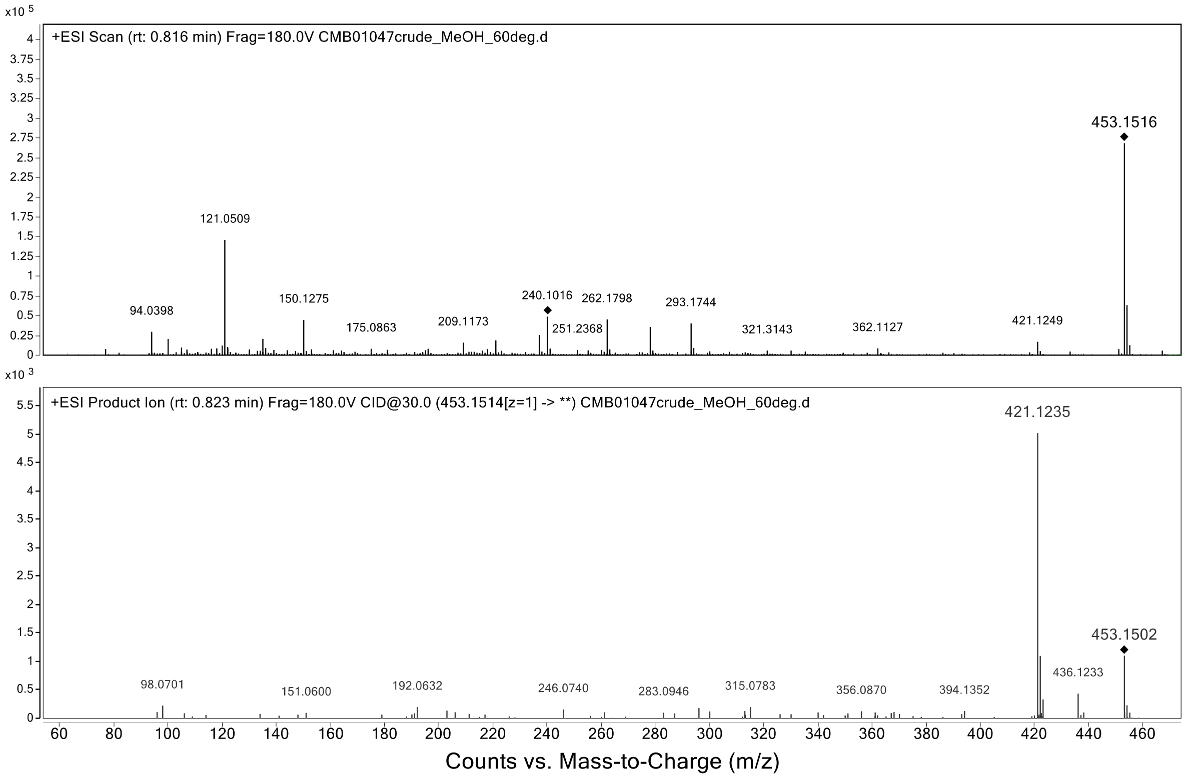

Figure S17. UHPLC-QTOF-MS/MS analysis of crude EtOH extract treated with MeOH at 60 °C, (top) +ESI TIC scan at t_R 0.816 min representing **1b** (*m/z* 453.1516); (bottom) MS/MS fragmentation for **1b** (loss of OMe resulting in *m/z* 421.1235).

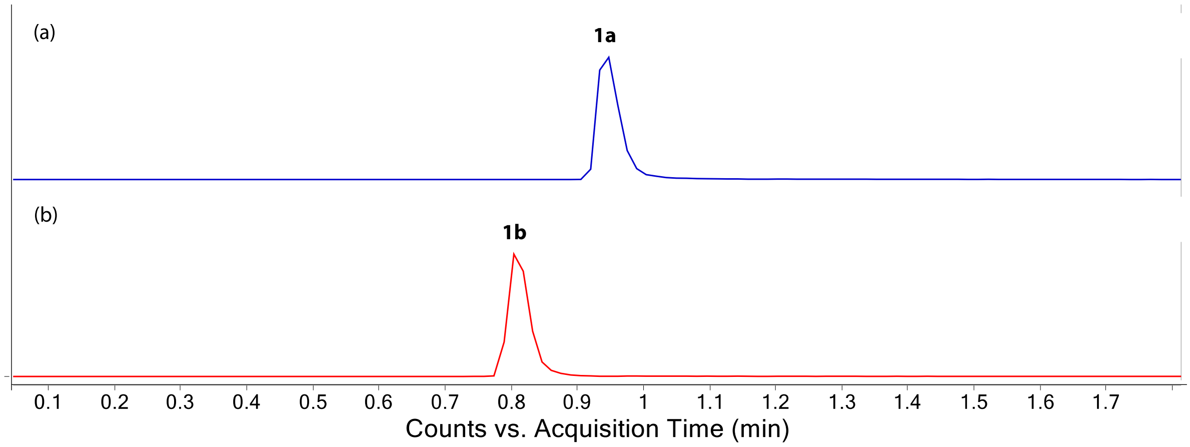

Figure S18. UHPLC-QTOF analysis of leucettazole A1 (**1a**) treated with MeOH at 40 °C. Single ion extraction (SIE) chromatograms for (a) *m/z* 467.17 (**1a**); (b) *m/z* 453.15 (leucettazole A2, **1b**)

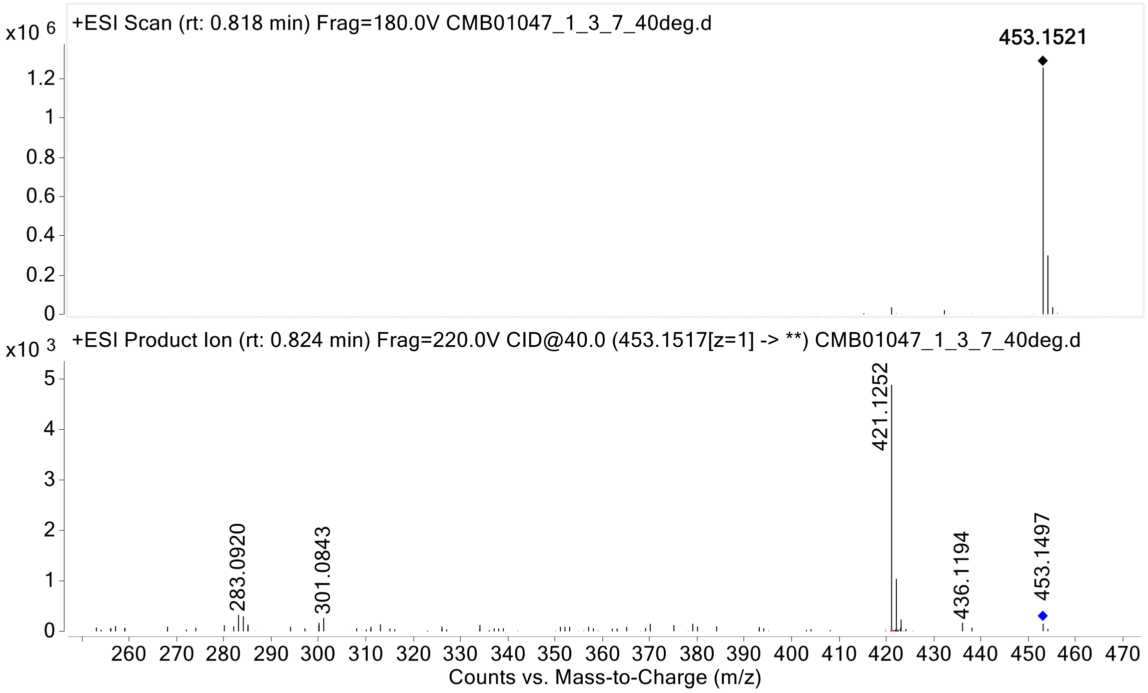

Figure S19. UHPLC-QTOF-MS/MS analysis of leucettazole A1 (**1a**) treated with MeOH at 40 °C; (top) +ESI TIC scan at t_R 0.818 min representing **1b** (*m/z* 453.1521); (bottom) MS/MS fragmentation for **1b** (loss of OMe resulting in *m/z* 421.1252).

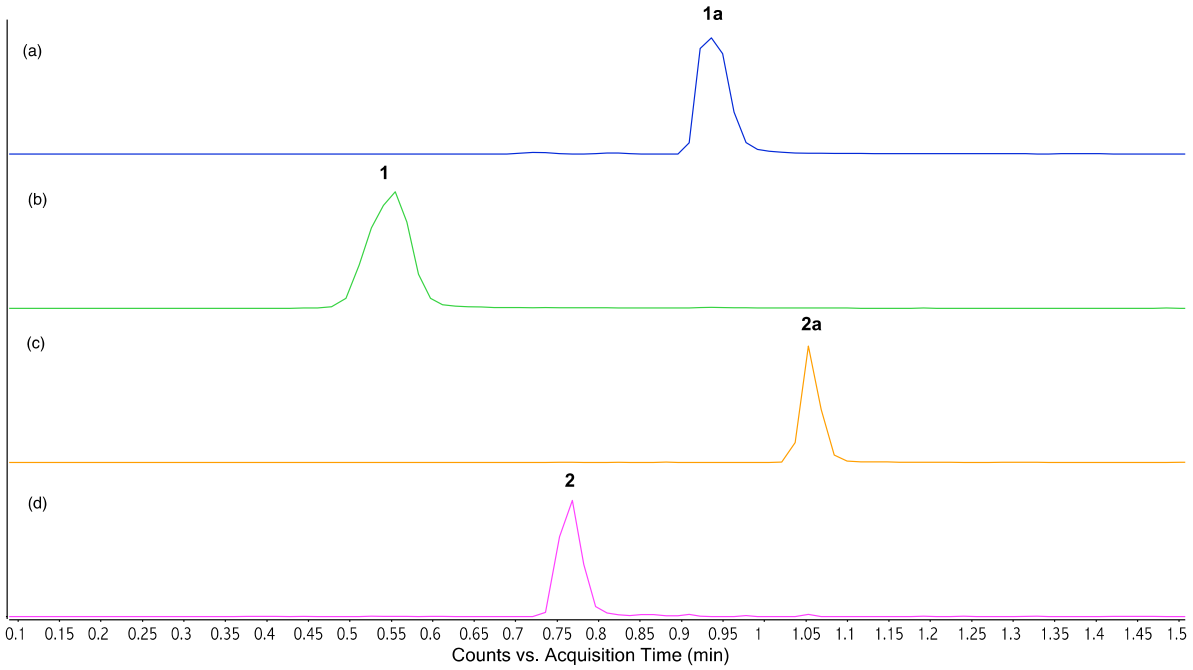

Figure S20. UHPLC-QTOF analysis of crude EtOH treated with aqueous MeCN. Single ion extraction (SIE) chromatograms for (a) *m/z* 467.17 (leucettazole A1, **1a**); (b) *m/z* 439.14 (leucettazole A, **1**); (c) *m/z* 481.18 (leucettazole B1, **2a**); (d) *m/z* 453.15 (leucettazole, **2**).

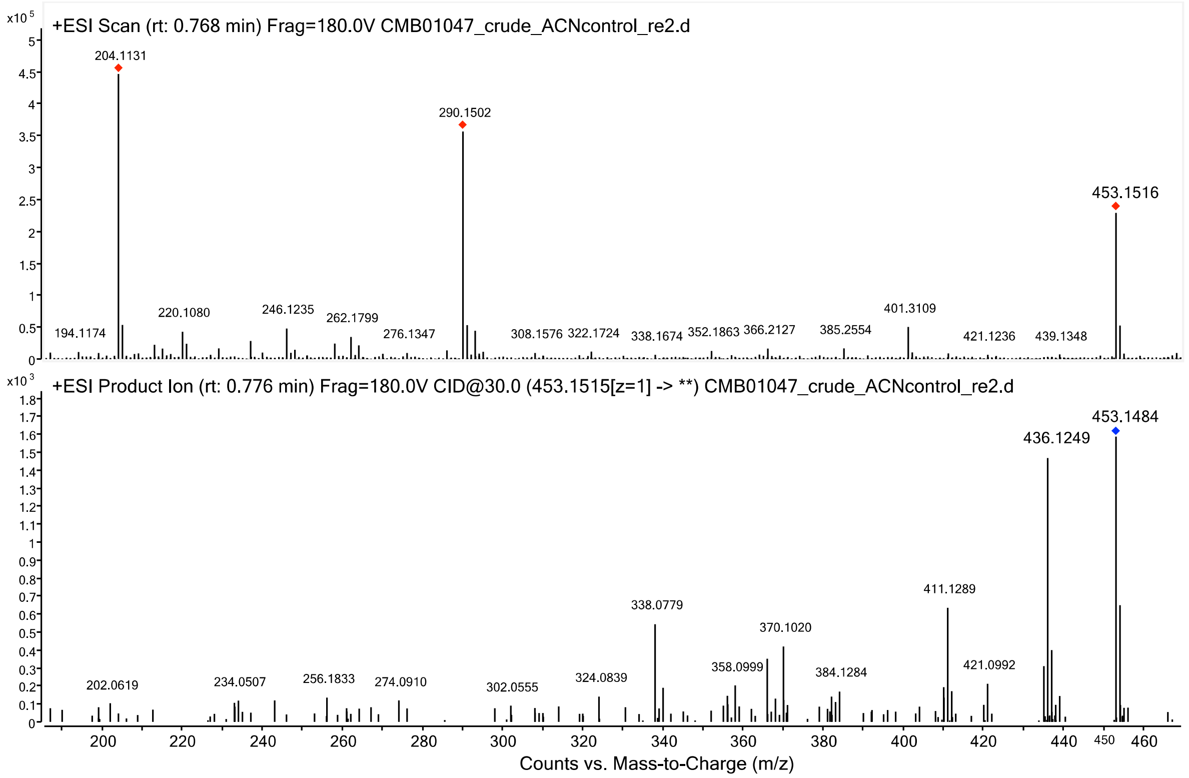

Figure S21. UHPLC-QTOF-MS/MS analysis of crude EtOH extract treated with aqueous MeCN, (top) +ESI TIC scan at t_R 0.768 min representing **2** (*m/z* 453.1516); (bottom) MS/MS fragmentation for **2** (loss of OH resulting in *m/z* 436.1249).

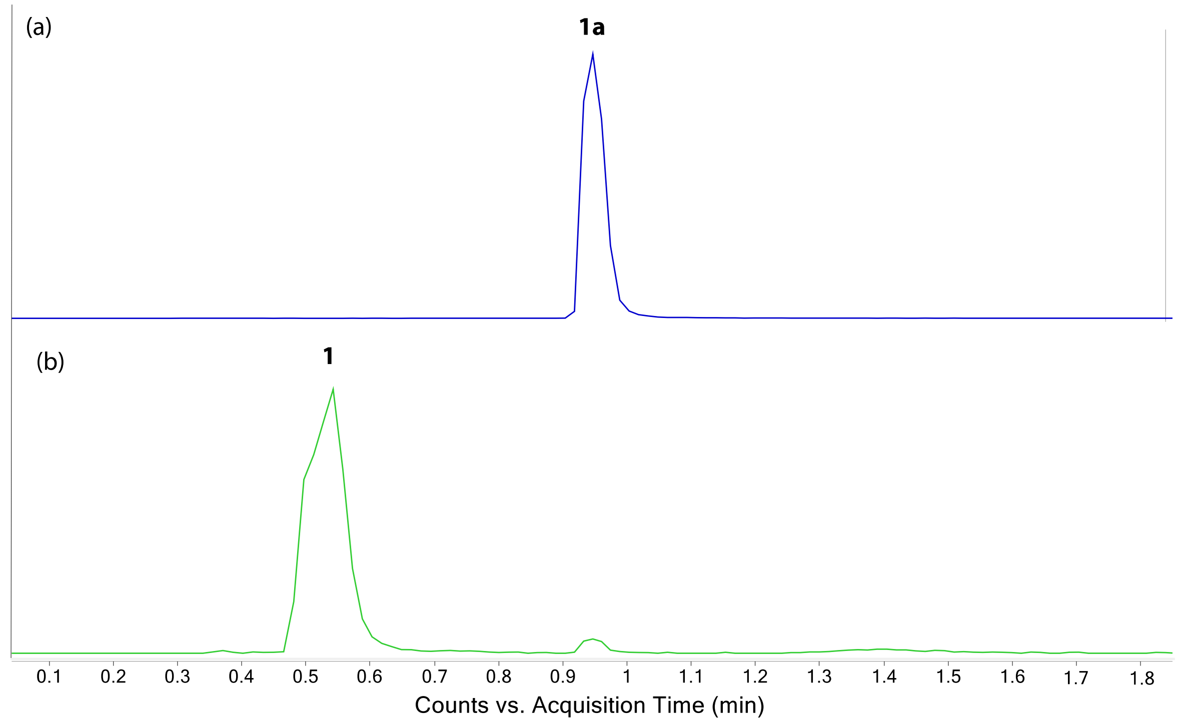

Figure S22. UHPLC-QTOF analysis of leucettazole A1 (**1a**) treated with 0.02% TFA/H_2O at 60 °C. Single ion extraction (SIE) chromatograms for (a) *m/z* 467.17 (**1a**); (b) *m/z* 439.14 (leucettazole A, **1**).

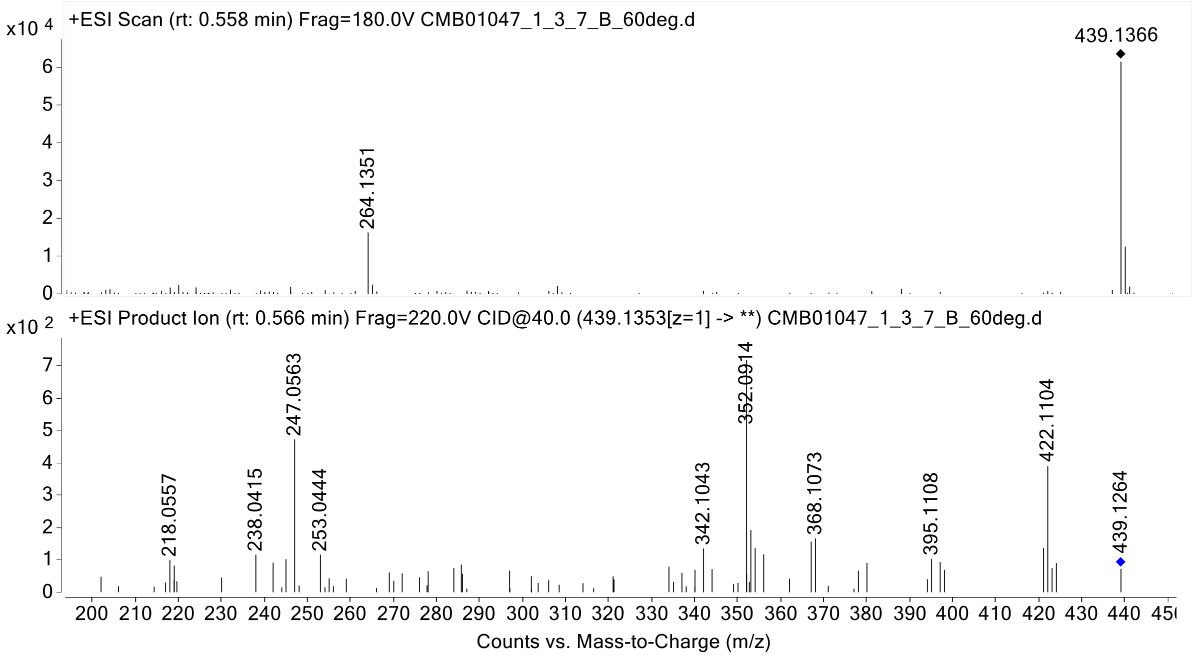

Figure S23. UHPLC-QTOF-MS/MS analysis of leucettazole A1 (**1a**) treated with 0.02% TFA/H_2O at 60 °C; (top) +ESI TIC scan at t_R 0.558 min representing **1** (*m/z* 439.1366); (bottom) MS/MS fragmentation for **1** (loss of OH resulting in *m/z* 422.1104).

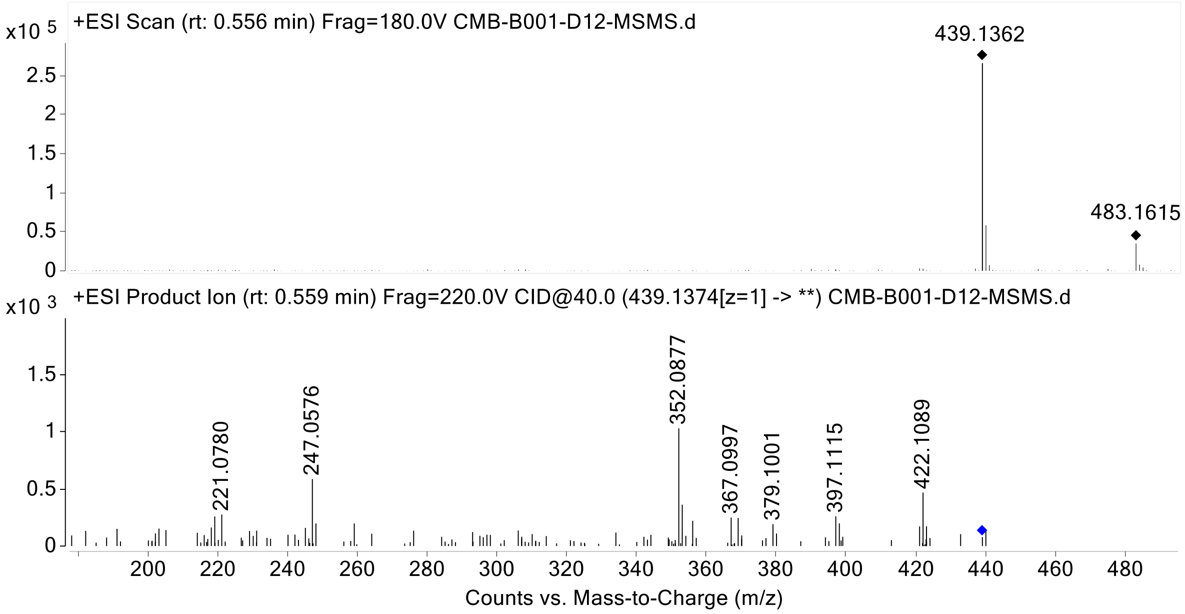

Figure S24. UHPLC-QTOF-MS/MS analysis of CMB-01047 *n*-BuOH solubles (top) +ESI TIC scan at t_R 0.556 min representing leucettazole A (**1**) (*m/z* 439.1362); (bottom) MS/MS fragmentation for **1** (loss of OH resulting in *m/z* 422.1089).

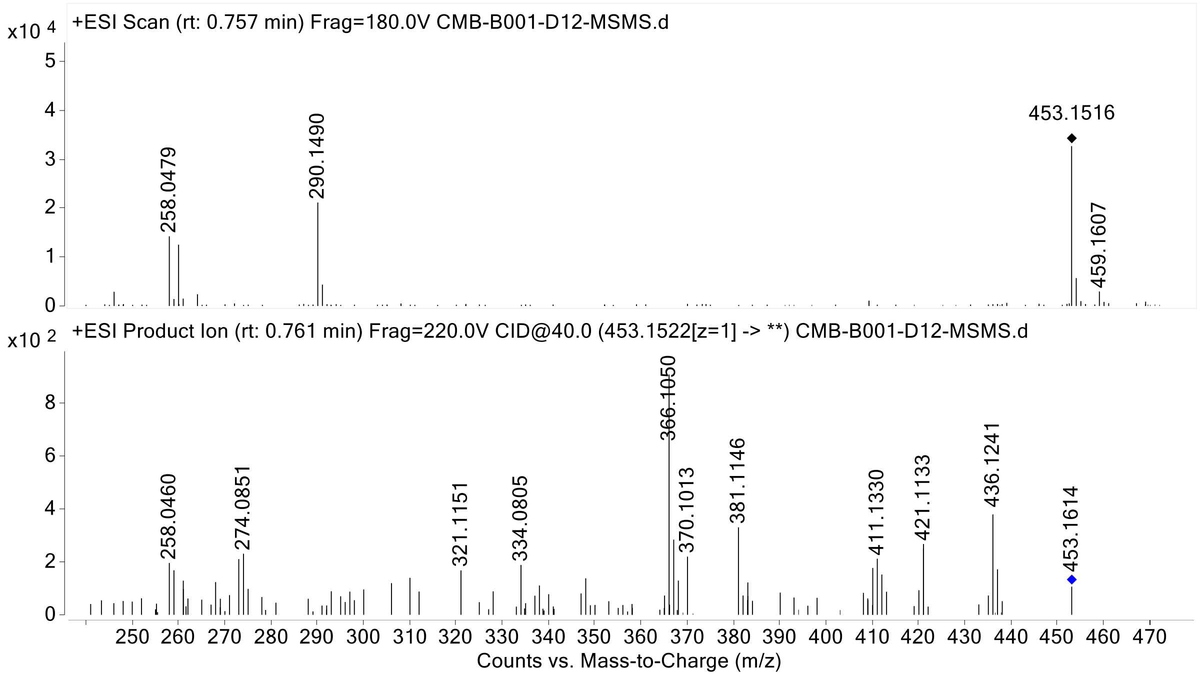

Figure S25. UHPLC-QTOF-MS/MS analysis of CMB-01047 *n*-BuOH solubles (top) +ESI TIC scan at t_R 0.757 min representing leucettazole B (**2**) (*m/z* 453.1516); (bottom) MS/MS fragmentation for **2** (loss of OH resulting in *m/z* 436.1241).

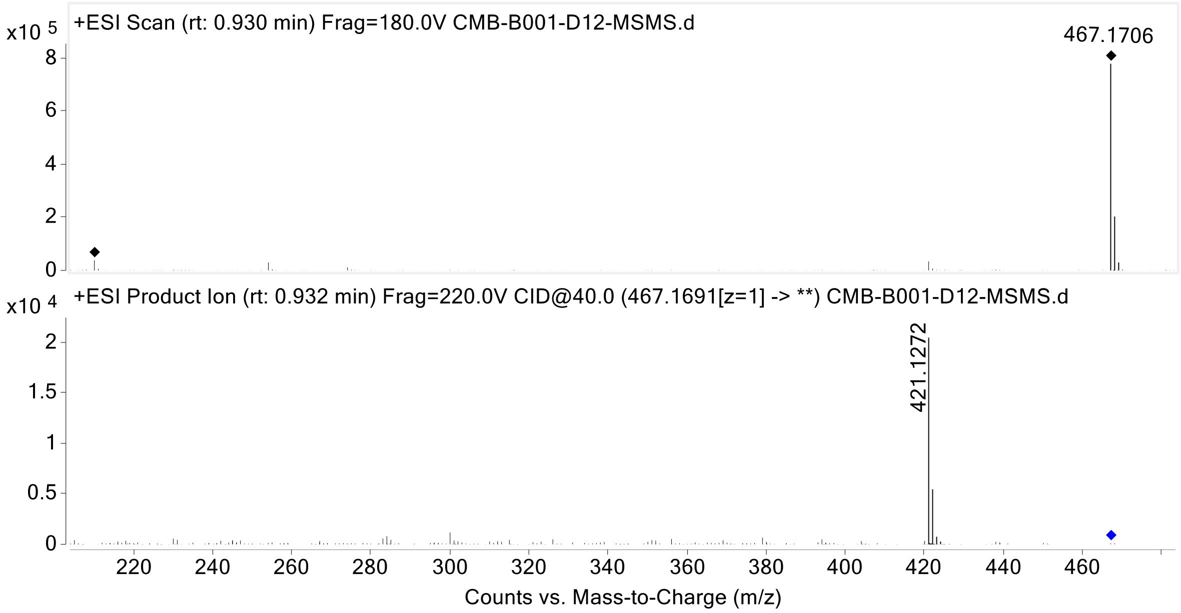

Figure S26. UHPLC-QTOF-MS/MS analysis of CMB-01047 *n*-BuOH solubles (top) +ESI TIC scan at t_R 0.930 min representing leucettazole A1 (**1a**) (*m/z* 467.1706); (bottom) MS/MS fragmentation for **1a** (loss of OEt resulting in *m/z* 421.1272).

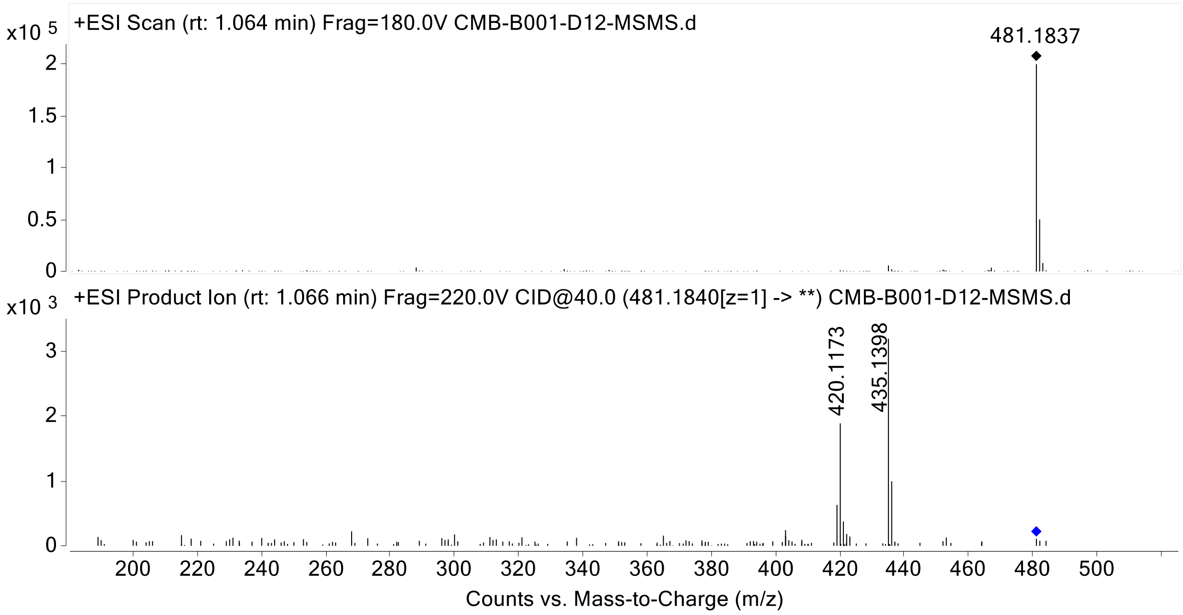

Figure S27. UHPLC-QTOF-MS/MS analysis of CMB-01047 *n*-BuOH solubles (top) +ESI TIC scan at t_R 1.064 min representing leucettazole B1 (**2a**) (*m/z* 481.1837); (bottom) MS/MS fragmentation for **2a** (loss of OEt resulting in *m/z* 435.1398).

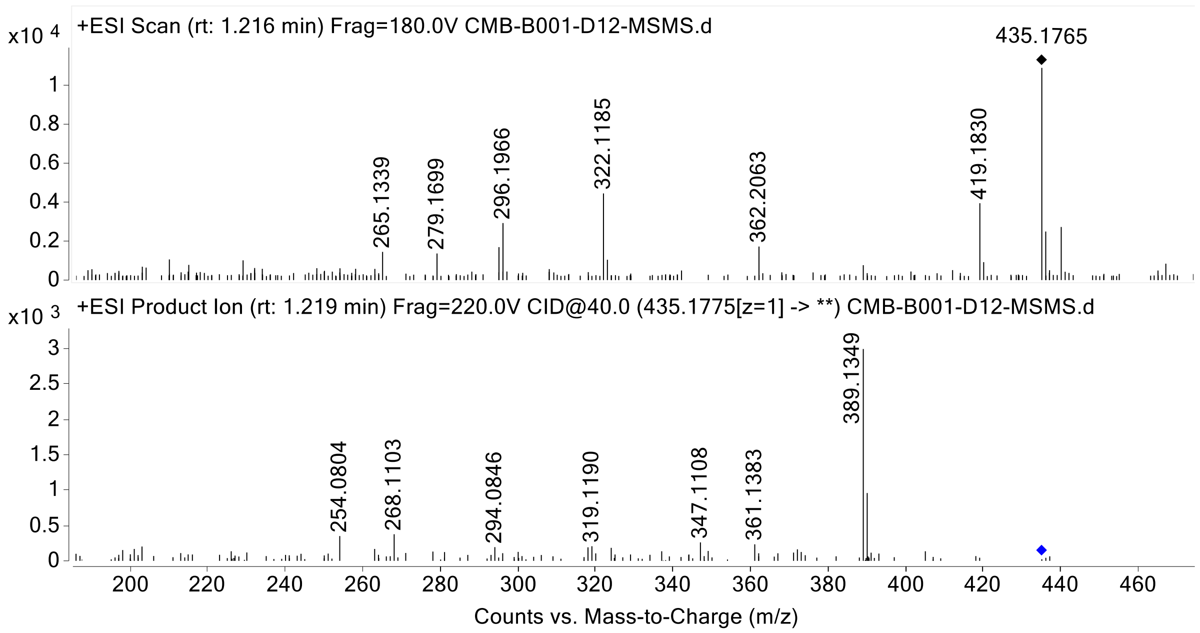

Figure S28. UHPLC-QTOF-MS/MS analysis of CMB-01047 *n*-BuOH solubles (top) +ESI TIC scan at t_R 1.216 min representing **i** (*m/z* 435.1765) (calcd for $C_{22}H_{23}N_6O_4^+$, 435.1775); (bottom) MS/MS fragmentation for **i** (loss of OEt resulting in *m/z* 389.1349).

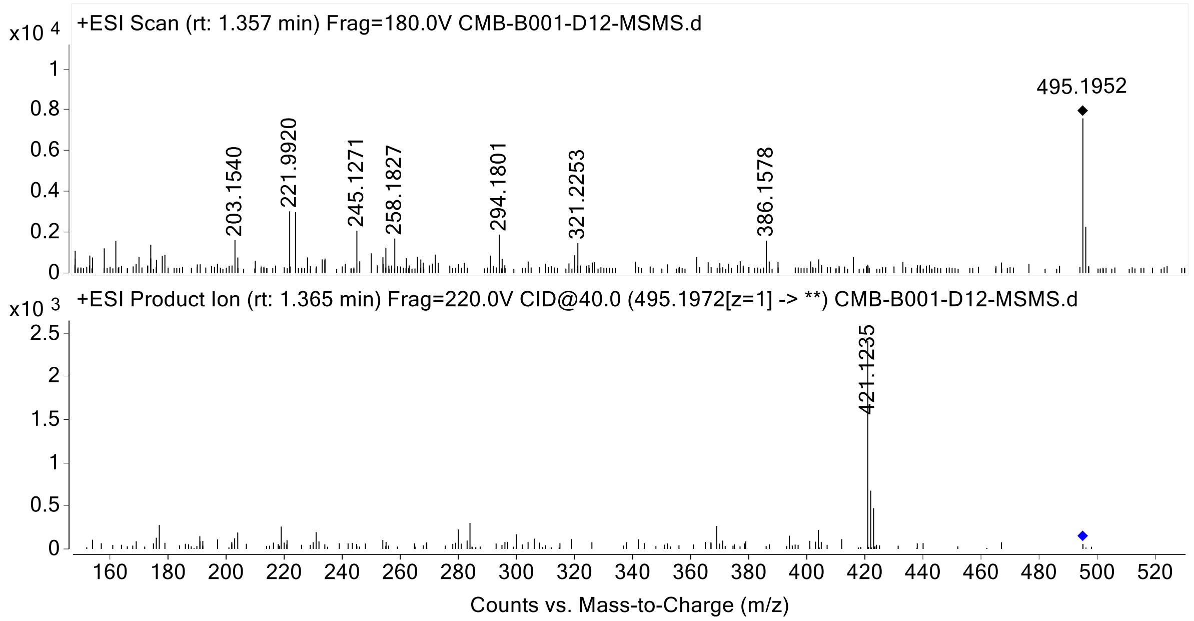

Figure S29. UHPLC-QTOF-MS/MS analysis of CMB-01047 *n*-BuOH solubles (top) +ESI TIC scan at t_R 1.357 min representing **ii** (*m/z* 495.1952) (calcd for $C_{24}H_{27}N_6O_6^+$, 495.1987); (bottom) MS/MS fragmentation for **ii** (loss of butyl ether resulting in *m/z* 421.1235).

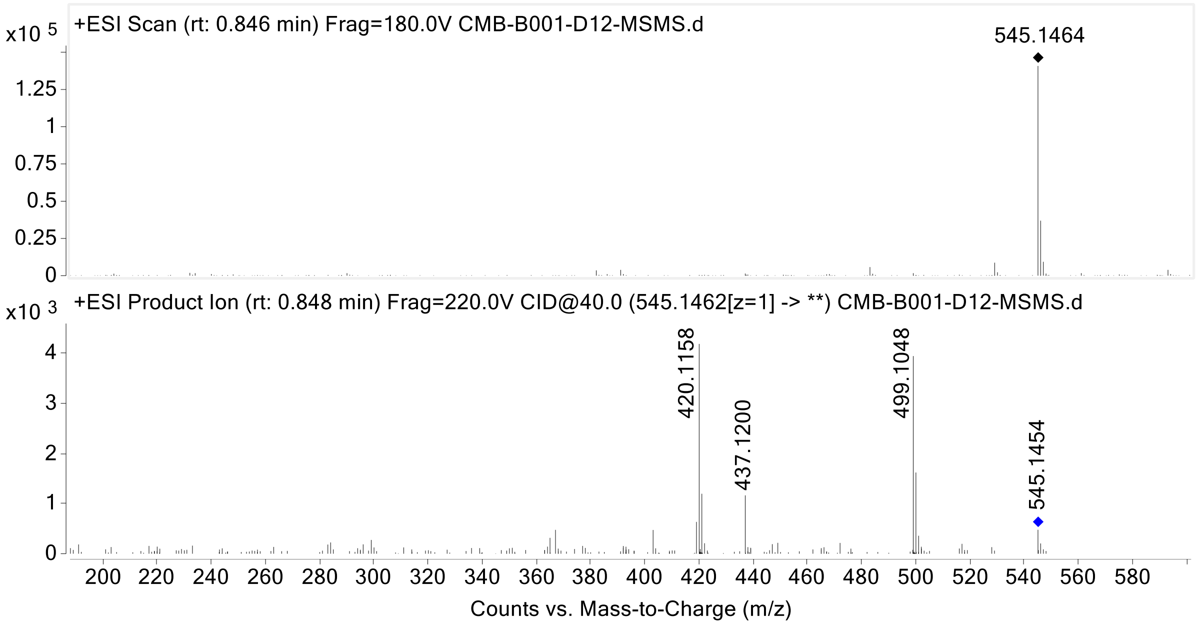

Figure S30. UHPLC-QTOF-MS/MS analysis of CMB-01047 *n*-BuOH solubles (top) +ESI TIC scan at t_R 0.846 min representing **iii** (*m/z* 545.1464) (calcd for $C_{23}H_{25}N_6O_8S^+$, 545.1449); (bottom) MS/MS fragmentation for **iii** (loss of EtOH resulting in *m/z* 499.1048).

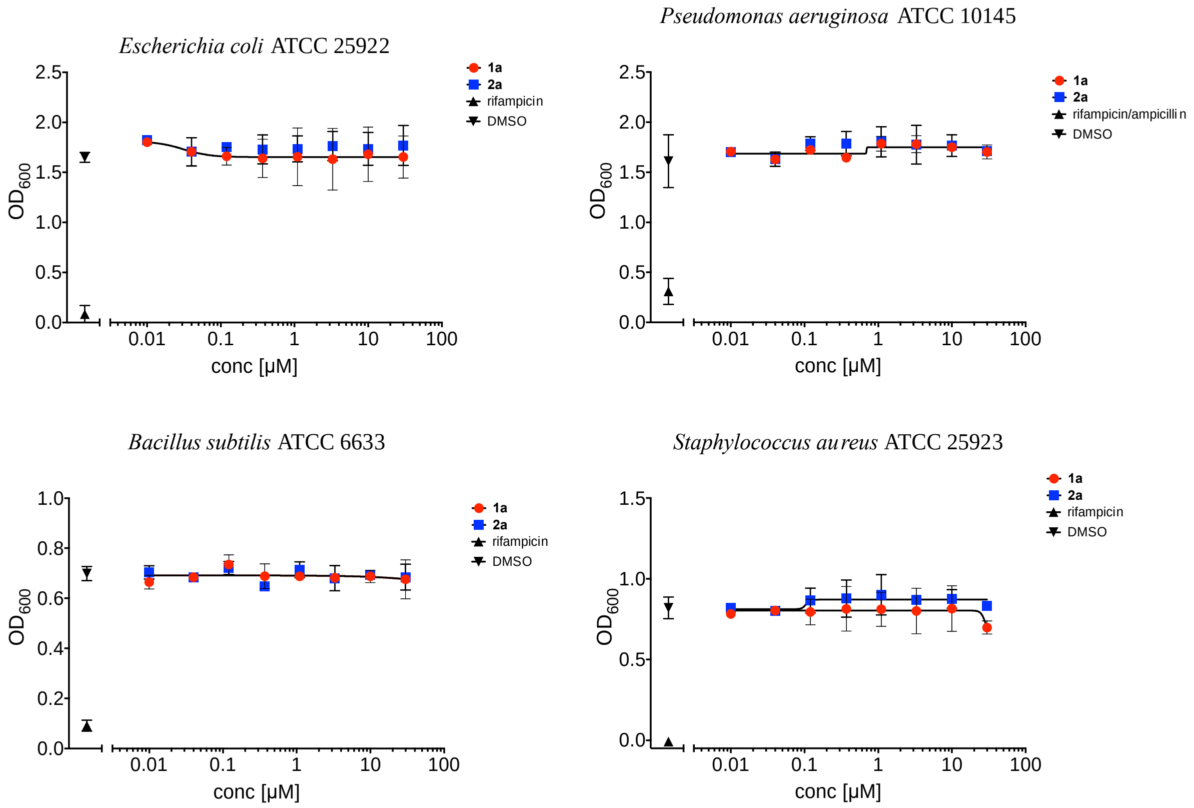

Figure S31. Antibacterial assay results for leucettazole A1 (**1a**) and leucettazole B1 (**2a**)

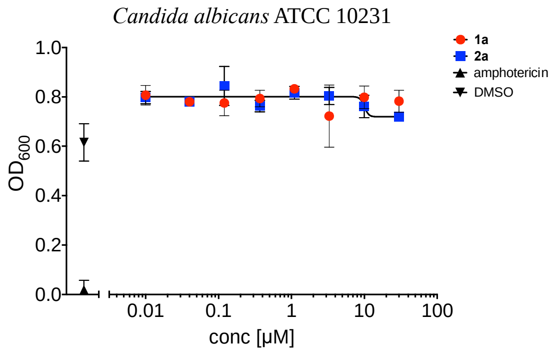

Figure S32. Antifungal assay results for leucettazole A1 (**1a**) and leucettazole B1 (**2a**)

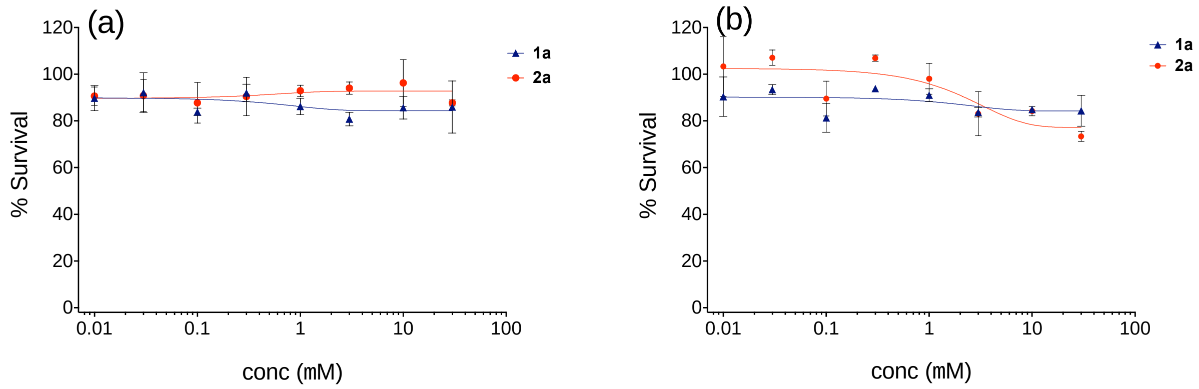

Figure S33. Cytotoxicity assay of leucettazole A1 (**1a**) and leucettazole B1 (**2a**) against (a) HEK293 (human embryonic kidney cell line) and (b) SW620 (human colon cancer cell line)
